# Adherence and acceptability of a robot-assisted Pivotal Response Treatment protocol for children with autism spectrum disorder

**DOI:** 10.1038/s41598-020-65048-3

**Published:** 2020-05-15

**Authors:** Iris van den Berk-Smeekens, Martine van Dongen-Boomsma, Manon W. P. De Korte, Jenny C. Den Boer, Iris J. Oosterling, Nienke C. Peters-Scheffer, Jan K. Buitelaar, Emilia I. Barakova, Tino Lourens, Wouter G. Staal, Jeffrey C. Glennon

**Affiliations:** 1 Department of Cognitive Neuroscience, Donders Institute for Brain, Cognition and Behaviour, Radboud University Nijmegen Medical Centre, P.O. Box 9104, 6500 HB Nijmegen, The Netherlands; 2 Karakter Child and Adolescent Psychiatry University Centre, Reinier Postlaan 12, 6525 GC Nijmegen, The Netherlands; 3 Karakter Child and Adolescent Psychiatry, Postbus 68, 6710 BB Ede, The Netherlands; 4 Behavioural Science Institute, Radboud University Nijmegen, PO Box 9104, 6500 HE Nijmegen, The Netherlands; 5 Driestroom, PO box 139, 6660 AC Elst, The Netherlands; 6 Faculty of Industrial Design, University of Technology, Eindhoven, P.O. Box 513 5600 MB Eindhoven, The Netherlands; 7 TiViPE, Kanaaldijk ZW 11, 5706 LD Helmond, The Netherlands; 8 Institute for Brian and Cognition, Leiden University, P.O. Box 9600 (C2-S), 2300 RC Leiden, Netherlands

**Keywords:** Autism spectrum disorders, Therapeutics

## Abstract

The aim of this study is to present a robot-assisted therapy protocol for children with ASD based on the current state-of-the-art in both ASD intervention research and robotics research, and critically evaluate its adherence and acceptability based on child as well as parent ratings. The robot-assisted therapy was designed based on motivational components of Pivotal Response Treatment (PRT), a highly promising and feasible intervention focused at training “pivotal” (key) areas such as motivation for social interaction and self-initiations, with the goal of establishing collateral gains in untargeted areas of functioning and development, affected by autism spectrum disorders. Overall, children (3–8 y) could adhere to the robot-assisted therapy protocol (Mean percentage of treatment adherence 85.5%), showed positive affect ratings after therapy sessions (positive in 86.6% of sessions) and high robot likability scores (high in 79.4% of sessions). Positive likability ratings were mainly given by school-aged children (*H*(1) = 7.91, *p* = .005) and related to the movements, speech and game scenarios of the robot. Parent ratings on the added value of the robot were mainly positive (Mean of 84.8 on 0–100 scale), while lower parent ratings were related to inflexibility of robot behaviour.

## Introduction

Digital technology can support interventions and care for children with psychiatric disorders^1^ and the development and use of new technologies within mental health care interventions is placed high on the international agenda^2^. Since demands from the social environment can be challenging and confusing for children with autism spectrum disorder (ASD)^3,4^ and their gains from high predictability, the use of technology within intervention such as robotics can be especially beneficial for them. Robots may be intrinsically appealing to children with ASD and contribute to their motivation for social interaction^5,6^ and the number of studies to the use of robots for children with ASD increased markedly during the last decade^7^.

Despite the assumed benefits, thus far, pilot studies do not show unambiguously positive results of using robots in interventions that target core symptoms (i.e. deficits in social communication) of children with ASD^8–12^. Also, heterogeneity in findings is reported in studies examining the potential value of adding a robot to existing interventions for ASD. In a study examining a 8-week robot-assisted intervention based on the principles of applied behaviour analysis (ABA), Treatment and Education of Autistic and related Communication-Handicapped Children (TEACCH) and the Picture Exchange Communication System (PECS), no increases in joint attention were found^13^. Moreover, although children showed more social attention in the robot-assisted intervention condition, this attention was predominantly not spontaneous and reduced during the mid and late sessions^13^. Another study showed that spontaneous question asking increased during a 6-session Social Story intervention with a robot^14^. In contrast, other studies found no significant changes in verbal initiations, responses and play behaviour during a 5-week intervention with a robot based on Lego© therapy^15^ or a similar increase in self-initiated question asking during a 4-session ABA-based intervention with a robot compared to a human trainer condition in children with ASD^16^. Results of these studies differ highly, which may reflect the use of different sample sizes and research methodology^17^. Furthermore, little is known about how children with ASD and their parents rate the acceptability of using a robot within existing interventions, with only one study reporting on this^15^. Specifically, parents of children with ASD reported that their child enjoyed the robot-assisted intervention and that components of the intervention where rated as acceptable by children with ASD^15^, although only 3 children with ASD were included in the study. Currently, no studies are available that elaborate on reasons for low or high acceptability and likability ratings during a robot-assisted intervention for children with ASD. Additionally, while treatment adherence is regarded as a core underlying assumption for evidence-ba sed interventions^18^ it is remarkable that earlier studies to newly developed protocols for robot assistance within established interventions for ASD do not report on this.

Furthermore, it is unclear how the design of the robot-assisted intervention relates to treatment adherence and acceptability of both children with ASD and their parents. Specific features of robot-assisted interventions may only be appropriate for subgroups of children with ASD, since a high heterogeneity in responses towards a robot exists also *within* small study samples of children with ASD; in eye contact, imitation, and touch behaviour^19^ and on approach and avoidance behaviour^20^. Although investigation of subgroups based on individual characteristics such as age, (verbal) IQ, severity of ASD symptoms and psychiatric comorbidity is key for a better understanding of which children with ASD benefit from robot-assisted interventions^14,21^ this has not been done in earlier studies.

In previous studies, specific design features are identified that may facilitate the appropriateness of robot-assisted interventions for children with ASD: 1) co-creation between researchers in psychopathology, neuroscience, robotics, and engineering in developing robot-assisted intervention protocols^22–24^, 2) using a robot within established treatment models for ASD^23,25^ and during multiple sessions^17^, 3) using a robot that is roughly the size of the child undergoing the therapy while balancing mechanical and human-like robotic features^26^, 4) providing input for robot behaviour by a human therapist^21,25^ while using movements and sounds within the robot’s behaviour for attracting the child’s attention and for use as positive reinforcements^26^, 5) incorporating child choice and a high level of adaptability to each specific child within the robot’s behaviour^23,24,26^, and 6) emphasizing, throughout the intervention, the possibility of generalization of skills learned during robot interaction to human interaction^17,25,27^. However, little is known about whether young children with ASD and their parents can adhere to a robot-assisted intervention protocol that incorporates these promising design features.

Here, we aim to present a robot-assisted Pivotal Response Treatment (PRT) protocol while exploring its treatment adherence and acceptability based on both child and parent ratings for subgroups of children with ASD. As a highly promising and feasible intervention, PRT aims at establishing collateral gains in untargeted areas of functioning and development by training “pivotal” (key) areas such as motivation for social interaction and self-initiations^28^. In our design we combine specific motivational components for children with ASD derived from both PRT and robotics research (see *Methods*). Incorporating these, we hypothesize that children with ASD will show high likability and affect scores towards the robot during a 20-session weekly intervention. Similarly, we anticipate that parental ratings on whether the robot has an added value to the PRT are high. Additionally, we aim to explore whether child and parent ratings of using a robot within PRT differ by individual characteristics of age, IQ, severity of ASD symptoms and psychiatric comorbidity. Lastly, reasons for child and parent ratings are explored to obtain a comprehensive view on which features of the robot-assisted intervention can be beneficial for children with ASD.

## Methods

### Participants

Twenty-five participants received robot-assisted PRT. This group was part of a larger randomised clinical trial to the effectiveness of PRT for children with ASD (PicASSo project, registered at 01/08/2014 at the Netherlands Trial Register; https://www.trialregister.nl/trial/4487; NL4487/NTR4712). Inclusion criteria were: 1) a clinical diagnosis of ASD, 2) meeting criteria for ASD based on DSM-IV^29^, 3) aged 3–8 years, 4) a total intelligence quotient (TIQ) of above 70, 5) ability to speak with one-word sentences at minimum, and 6) at least one of the parents speaks Dutch to the child. Additionally, an exclusion criterion was having received PRT previously. Dosages of medication were stable at start of the intervention phase and maintained so as much as possible during the intervention. Of the 25 participants, 23 (92%) scored above the cut-off for ASD on the Autism Diagnostic Observation Schedule (ADOS-2)^30^ while two participants scored one point below the cut-off. These participants were included in the study as they had a very clear clinical ASD diagnosis, based on a thorough multidisciplinary and multi-informant psychiatric examination. For mothers and fathers of participants respectively, education level was low for 24% and 27%; average for 36% and 23%, and high for 40% and 50%.

### Procedures

Participants were recruited from clinical referrals to the outpatient departments of Karakter, an expert center for child and adolescent psychiatry in The Netherlands. Parents received verbal and extensive written information on the outline and aims of the study and both parents signed an informed consent form prior to inclusion. Eventual missing baseline measures were administered if these had not been administered as part of the diagnostic procedure (see *Measures*). Prior to the robot-assisted PRT, parents received psycho-education on ASD if not received in the past, either individually or group-based. Robot-assisted PRT consisted of 20 sessions, once a week, by a certified PRT-therapist trained up until level III. The study received ethical approval by the Dutch Research Ethical Committee (CMO Arnhem-Nijmegen, NL50509.091.14) and all procedures were in accordance with the 1964 Helsinki declaration and its later amendments.

### Intervention protocol

The robot-assisted intervention was based on the treatment manual that was developed by the original PRT developers^31^ and translated into Dutch. Within the study, a 20-session intervention protocol was used that involved training children pivotal behaviours such as motivation for social interaction, self-initiations and multiple cues and training parents in using the PRT techniques at home. Before the initial PRT session, the therapist discussed the treatment procedures with parents, while child target behaviours were discussed. There were 14 parent-child sessions, in which the parents practiced the PRT techniques during parent-child interaction. Four sessions were parent sessions, in which the progress of the child was discussed, as well as the parental use of the PRT techniques at home. In 2 sessions the child’s teacher (or day care attendant) was involved to discuss and practice the use of the PRT techniques at school/daycare. The sessions were held weekly, with two parent-child sessions followed by one parent-only session throughout the intervention. Each PRT session had a duration of 45 minutes, except for one 90-min visit for PRT implementation within the class- or day-care room.

A NAO robot, controlled by the PRT therapist, was used in the first 15–20 minutes of each of the 14 parent-child sessions. Parents were seated close to the child and were asked to observe how the PRT techniques were used to improve their child’s communication. In this way, the therapist was able to model the PRT techniques for the parents by controlling the robot. Although the game scenarios were primarily designed for robot-child interaction, the parent was instructed to directly and naturally reward the child when he or she showed an appropriate initiation towards the parents. After the robot-assisted part of the session, parents practiced the PRT techniques during a game with their child and the therapist provided feedback during and after the session.

The following PRT motivational techniques^32^ were used in the development of game scenarios for robot-child interaction:

#### Child choice

Nine different game scenarios for robot-child interaction were created that are developmentally appropriate for children aged 3–8 years. Based on parental information and child’s preferences during the therapy session(s), the type of game (i.e. puzzles, Lego©, or cards) was chosen. The kind of game (i.e. type of either puzzle, or Lego©, or cards) was chosen by the child during the interaction with the robot. Furthermore, a text-to-speech module could be activated when the child changed the subject of the robot-child conversation.

#### Child attending and providing a clear opportunity to respond

Learning opportunities were included in the game scenarios for robot-child interaction by 1) placing the desired materials in a closed box before start of the game, 2) providing the child with only parts of the game materials at once, and 3) providing only parts of information about a game. Learning opportunities were provided if the child was interested in the robot and the game. If the child’s attention was drawn to another game or subject, the therapist used the text-to-speech module to respond accordingly.

#### Interspersing maintenance tasks

A game scenario was selected that included learning opportunities for both maintained (easy) and new (difficult) tasks for each child. Nine different therapeutic game scenarios were created, each with seven different levels of complexity. The level of prompting (i.e. the help that the child received for showing appropriate behaviour) could be adjusted throughout the game scenario and interspersed between easy (e.g. tell prompt) and difficult (e.g. wait prompt).

#### Direct and natural reinforcement

In the pre-programmed game scenarios, direct and natural reinforcement was provided upon the child’s behaviour. For instance, when the child asked: “robot, can you open the box?” the therapist directly controlled the robot in opening the box. Also, when a child took an initiative that was not anticipated in the pre-programmed game scenario, the therapist used the text-to-speech module to provide a direct and natural reinforcement.

#### Reinforcement of attempts

When the child showed the target behaviour or an appropriate attempt, a direct and natural reinforcement was provided by the robot (i.e. the therapist controlling the programming environment). However, when the child did not initiate spontaneously or the attempt was deemed inappropriate, the the text-to-speech module was activated to prompt the child.

Information on the type of robot and controlling of robot behavior and a detailed description on the development of game scenarios for robot-child interaction is described in the Supplementary Information.

### Measures

#### Initial measures

Demographic information on participant’s age of inclusion, gender, and psychiatric comorbidity was extracted from case files. Estimating total intelligence quotient (TIQ) was based on either the Wechsler Intelligence Scale for Children (WISC-III)^33^ Wechsler Preschool and Primary Scale of Intelligence (WPPSI-III)^34^ or Mullen Scales of Early Learning (MSEL)^35^.

Severity of ASD symptoms was assessed by initial administration of the ADOS-2^30^, a semi-structured observation schedule measuring social communication and repetitive, restricted behaviour. An ADOS-2 severity score was calculated^36^. Scores 1–4, 5–7 and 8–10 represented low, moderate and high severity of ASD symptoms respectively.

#### Treatment adherence

Treatment adherence was defined as the percentage of therapy sessions in which both the child and parents accepted the use of the NAO robot and in which the child showed sufficient motivation to complete the 15- to 20-min robot-child interaction. For participants that were unable to complete the protocol due to reasons that were unrelated to the use of the NAO robot (e.g. because of another indication for intervention), the percentage was calculated over the number of sessions that were received. Reasons for incomplete treatment adherence were documented.

#### Child affect and likability of robot

At the start and end of each robot-assisted session, the child was presented with a 5-point Visual Analogue Scale (VAS) measuring child affect (i.e. ‘how happy are you now?’, indicated with smilies). At the end of the session child likability of the robot (i.e. ‘did you like the robot today?’, indicated with thumps up and down) was measured. Additionally, when possible, the child was asked to indicate a reason for their affect and likability ratings. Reasons for child affect ratings were categorized into (1) related to the robot’s movements and/or speech, (2) related to the game play with the robot, (3) related to the therapy session, but not to the robot (e.g. game play with parents), (4) not related to therapy session, (5) no reason stated. Percentages were calculated for negative (score 1 or 2), neutral (score 3) and positive (score 4 and 5) affect ratings. Reasons for robot likability by the child were categorized into: (1) related to robot appearance, (2) related to robot’s movements and/or speech, (3) related to the game play with the robot, (4) not directly related to the robot, (5) no reason stated. Percentages were calculated for negative (score 1 or 2), neutral (score 3) and positive (score 4 and 5) robot likability ratings.

#### Parent ratings of robot-assisted sessions

Additional to the child ratings, parents were asked to complete the Session Rating Scale (SRS)^37^ after each robot-assisted parent-child session. The SRS has been developed for clinical use during different interventions and has been shown to have a high reliability (Cronbach’s α = .93) and moderate concurrent validity (range of *r* = .37-.63 with Working Alliance Inventory)^38^. On a VAS line ranging from 0 to 100, parents indicated 1) whether they felt heard, understood and respected (*Relationship*), 2) whether the session worked on what parents wanted to work on (*Goals and Topics*), 3) whether the therapist’s approach was a good fit (*Approach or Method*) and 4) overall, whether today’s session was right for the parent (*Overall*). For the purpose of this study, two VAS lines ranging from 0 to 100 were added to the SRS which were the main focus of this study: 1) whether the communication of the robot towards the child was clear (*Robot Communication*) and 2) whether the robot was an additional value to the current therapy session (*Robot Value*). Higher scores on the VAS reflected a more positive attitude of parents.

### Ethical approval

All procedures performed in studies involving human participants were in accordance with ethical standards of the institutional and national research committee and with the 1964 Helsinki declaration and its later amendments or comparable ethical standards.

### Informed consent

Informed consent was obtained from all individual participants included in the study.

## Results

To explore whether child and parent ratings of using a robot within PRT differ by individual characteristics, subgroups of participants were made based on age (pre-school: 3–5 years versus school-aged: 6–8 years), gender (male versus female), TIQ (below average: <90, versus average: 90–109, versus above average: > 109), severity of ASD symptoms (low versus moderate versus high severity) and psychiatric comorbidity (co-morbid diagnosis of attention-deficit hyperactivity disorder (ADHD) versus other co-morbid psychiatric disorder(s), versus no co-morbidity).

### Descriptive statistics and treatment adherence

Table 1 shows descriptive statistics and percentages of treatment adherence for the total group and for subgroups of participants based on individual child characteristics with results of exploratory non-parametric Kruskal-Wallis tests for between-group differences. Overall, the mean percentage of treatment adherence was high and there were no differences in percentages treatment adherence between the groups based on individual child characteristics (see Table 1). The majority of participants showed either complete adherence with the robot-assisted PRT protocol (34,8%) or did not show adherence for (only) 1 session (26,1%) or 2 sessions (26,1%) of the total number of sessions. Main reasons for incomplete adherence were: 1) game scenario was inadequately adjusted to the interests and/or skills of the child, 2) child lost interest for the robot-assisted game scenarios during later sessions, 3) child showed anxiety towards the robot during the first session, and 4) technical problems with robot hardware and/or software. Exploration of outliers in the total group indicated that for three of the 25 participants (12%), treatment adherence was below 2 SDs of the mean.

**Table 1:** Descriptive Statistics of Treatment Adherence for Total Group and Subgroups of Participants based on Individual Characteristics and Results of Exploratory Analyses.

Total group	Descriptive Statistics		Treatment Adherence		
	*M* (*SD*)	*N* (% of Total)	*M* (*SD*)	*H* (df)	*p*
		25	85.5% (17.5)		
Age	6.2 (1.3)			0.04 (1)	.835
Preschool		8 (32.0%)	91.1% (10.5)		
School-aged		17 (68.0%)	86.2% (15.4)		
Gender				0.39 (1)	.532
Male		20 (80.0%)	89.2% (12.6)		
Female		5 (20.0%)	81.1% (19.4)		
Total IQ	101.8 (14.2)			0.59 (2)	.743
Below average		3 (12.0%)	86.8% (11.9)		
Average		14 (56.0%)	88.8% (13.9)		
Above average		6 (24.0%)	84.5% (17.7)		
Missing		2 (8.0%)			
ADOS severity	6.0 (1.5)			3.68 (2)	.159
Low		3 (12.0%)	83.3% (28.9)		
Moderate		17 (68.0%)	85.5% (12.5)		
Severe		5 (20.0%)	95.6% (6.5)		
Psychiatric comorbidity				1.80 (2)	.407
AD(H)D		3 (12.0%)	92.9% (7.1)		
Other		2 (8.0%)	96.2% (5.4)		
None		20 (80.0%)	85.6% (15.4)		

*Note*. AD(H)D = attention deficit (hyperactivity) disorder, ADOS = autism diagnostic observation schedule, df = degrees of freedom, H = test statistic resulting from Kruskal-Wallis test, IQ = intelligence quotient, M = mean, N = number of participants, p = p-value, SD = standard deviation.

Reasons for aborting the use of the robot in these participants were either that the participant rejected the game scenario’s with the robot when noticing that the therapist controlled robot’s behaviour (2 participants), or that the participant showed anxiety towards the robot during multiple sessions (1 participant).

### Child and parent ratings

Table 2 shows the mean child affect and likability ratings for the total group and for subgroups based on individual characteristics, with results of the exploratory Kruskal-Wallis tests for between-group differences. For the total group and for most subgroups based on individual characteristics, mean child affect and likability ratings were (very) positive, between 4 (“happy”/“I liked the robot”) and 5 (“very happy”/“I very much liked the robot”). No significant differences in child affect before the session where found between the groups based on individual characteristics, but a significant difference was found on child affect after the session for groups based on ASD severity (see Table 2). Specifically, a significant difference was found between the moderate and high ASD severity group (with the high severity group showing lower affect scores) (*H*(1) = 4.54, *p* = .033), but not between the low and moderate ASD severity group (*H*(1) = 2.74, *p* = .098), and between the low and high ASD severity group (*H*(1) = 0.56, *p* = .456). Furthermore, the preschool-aged group had lower mean robot likability scores compared with the school-aged group (see Table 2). Additionally, further exploration of robot likability ratings by preschool-aged participants indicated that 57.5% were positive, 29.9% were neutral and 12.6% were negative, while 90.9% of ratings of school-aged participants were positive; (only) 6.9% were neutral and 2.3% were negative.

**Table 2:** Child Affect and Likability Ratings and for the Total Group and Subgroups of Participants based on Individual Child Characteristics and Results of Exploratory Analyses.

Total group	*M*(*SD*)	*H* (df)	*p*	*M*(*SD*)	*H* (df)	*p*	*M*(*SD*)	*H* (df)	*p*
	4.3 (0.6)			4.5 (0.5)			4.3 (0.8)		
Age		1.72 (1)	.189		1.58 (1)	.208		7.91 (1)	.005**
Preschool	3.9 (0.8)			4.1 (0.5)			3.5 (0.8)		
School-aged	4.5 (0.5)			4.5 (0.5)			4.7 (0.4)		
Gender		1.85 (1)	.174		0.26 (1)	.609		0.23 (1)	.629
Male	4.2 (0.6)			4.4 (0.5)			4.3 (0.8)		
Female	4.7 (0.3)			4.2 (0.7)			4.5 (0.6)		
Total IQ		2.59 (2)	.274		0.26 (2)	.879		0.21 (2)	.899
Below average	4.8 (0.2)			4.5 (0.1)			4.5 (0.5)		
Average	4.2 (0.7)			4.4 (0.4)			4.3 (0.8)		
Above average	4.4 (0.5)			4.2 (0.9)			4.3 (0.9)		
ADOS severity		1.83 (2)	.401		6.23 (2)	.044*		2.06 (2)	.357
Low	4.3 (0.6)			3.6 (0.9)			4.2 (1.1)		
Moderate	4.4 (0.6)			4.6 (0.4)			4.5 (0.8)		
Severe	4.1 (0.6)			4.2 (0.3)			4.1 (0.4)		
Psychiatric comorbidity		0.77 (2)	.681		1.48 (2)	.476		0.70 (2)	.706
AD(H)D	4.4 (0.7)			4.3 (1.0)			4.3 (1.2)		
Other	4.2 (0.3)			4.1 (0.3)			4.1 (0.3)		
None	4.3 (0.6)			4.4 (0.5)			4.4 (0.8)		

*Note*. *p < .05, **p < .01, AD(H)D = attention deficit (hyperactivity) disorder, ADOS = autism diagnostic observation schedule, df = degrees of freedom, H = test statistic resulting from Kruskal-Wallis test, IQ = intelligence quotient, M = mean, p = p-value, SD = standard deviation.

Similarly as parent ratings on *Robot Communication* and *Robot Value* (see Table 3) mean parent ratings on the other scales of the SRS were high for the total group; 88.8 (*SD* = 7.7) on *Relationship*; 87.9 (*SD* = 8.3) on *Goals and Topics*; 88.3 (*SD* = 9.0) on *Approach or Method*; and 90.2 (*SD* = 7.6) on the *Overall* scale. Table 3 shows that parent ratings on *Robot Communication* and *Robot Value* were also high in each subgroup. Of *Robot Communication* ratings, 95.0% were above 50 (indicating “robot communicated clearly to the child”) and of *Robot Value* ratings, 94.3% were above 50 (indicating “robot was of additional value to the therapy session”). There were no significant differences on parent ratings of *Robot Communication* between subgroups based on individual child characteristics. However, in the subgroup of females, parents showed significantly higher mean ratings of *Robot Value* compared with the subgroup of males (see Table 3).

**Table 3:** Parent Ratings of Robot Communication and Additional Value of Robot for Total Group and Subgroups of Participants based on Individual Child Characteristics and Results of Exploratory Analyses.

Total group	Robot communication			Robot value		
	*M*(*SD*)	*H* (df)	*p*	*M*(*SD*)	*H* (df)	*p*
	83.6 (12.6)			84.8 (13.4)		
Age		0.22 (1)	.641		0.67 (1)	.415
Preschool	80.8 (15.4)			81.5 (13.8)		
School-aged	84.9 (11.3)			87.0 (11.8)		
Gender		2.66 (1)	.103		3.88 (1)	.049*
Male	81.5 (12.7)			83.1 (12.8)		
Female	92.3 (5.8)			94.2 (3.6)		
Total IQ		0.55 (2)	.761		0.71 (2)	.700
Below average	88.5 (10.0)			89.8 (9.5)		
Average	83.5 (12.2)			84.5 (12.8)		
Above average	82.2 (14.8)			85.8 (13.8)		
ADOS severity		1.48 (2)	.478		1.34 (2)	.513
Low	82.5 (23.7)			83.7 (21.2)		
Moderate	86.0 (8.8)			88.3 (8.3)		
Severe	78.0 (14.6)			78.2 (16.2)		
Psychiatric comorbidity		0.88 (2)	.643		0.04 (2)	.980
AD(H)D	79.3 (22.2)			82.5 (20.7)		
Other	78.7 (16.2)			78.4 (21.9)		
None	85.2 (10.6)			86.8 (10.4)		

*Note*. *p < .05, **p < .01, AD(H)D = attention deficit (hyperactivity) disorder, ADOS = autism diagnostic observation schedule, df = degrees of freedom, H = test statistic resulting from Kruskal-Wallis test, IQ = intelligence quotient, M = mean, p = p-value, SD = standard deviation.

Exploring reasons for high ratings indicated that parents: 1) noticed that the child was enthusiastic and motivated for the game scenario with the robot, 2) noticed that the child initiated at home and at school by telling about the robot. Furthermore, exploration of outliers indicated that 9.5% of parent ratings were below 2 SDs of the mean on *Robot Communication* and 8.0% of parent ratings were below 2SDs of the mean on *Robot Value*. Reasons for lower parent rating scores could be categorized in: 1) parents noticed that the child could not fully understand the robot speech (*Robot Communication*), 2) parents noticed that there was a delay in robot’s response towards the child (*Robot Communication*) and 3) parents noticed that the game scenarios were either too easy or too difficult (*Robot Value*).

### Reasons for child affect and likability ratings

**Figure 1:**
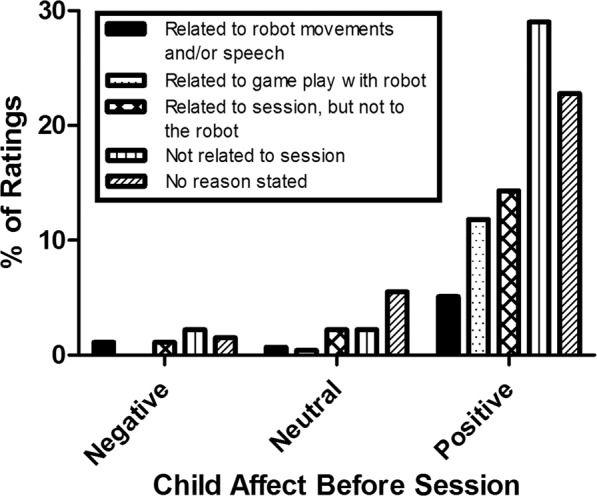
Reasons for child affect ratings before the robot-mediated therapy sessions, displayed separately for negative, neutral and positive ratings.

**Figure 2:**
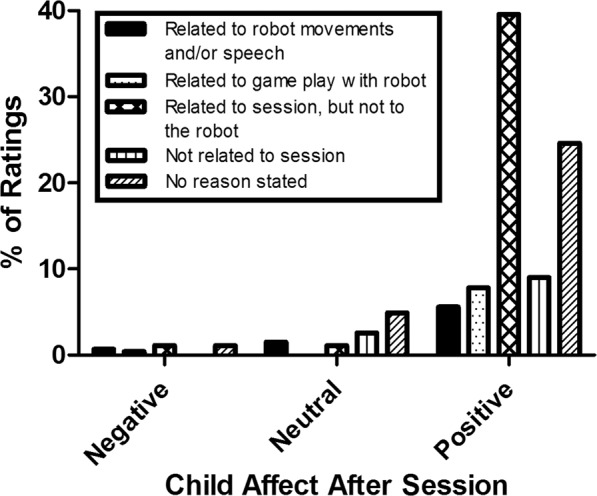
Reasons for child affect ratings after the robot-mediated therapy sessions, displayed separately for negative, neutral and positive ratings.

The majority of child affects ratings were positive, both before the session (83.1%, see Fig. 1) and after the session (86.6%, see Fig. 2). The majority of reasons for positive affect scores before the session were either not directly related to the therapy session (35.0%) or no reason for the positive rating was stated by the child (27.4%). Of the affect scores before the session, 11.0% were neutral and 5.9% were negative and most participants either did not state a reason or reasons were not related to the therapy session.

In contrast to ratings before the session, positive child affect ratings after the session were mostly related to the therapy session, however not specifically to the robot (45.7%). Of the positive affect ratings after the session, 6.5% was related to the movements and/or speech of the robot and 9.0% was related to the game play with the robot. Few affect ratings after the session were either neutral (10.1%) or negative (3.4%). Similar to the reasons for positive affect scores, few reasons for neutral or negative affect scores were directly related to robot movements and/or speech (14.8% of neutral ratings; 22.2% of negative ratings) or the game play with the robot (0% of neutral ratings; 11.1% of negative ratings).

**Figure 3:**
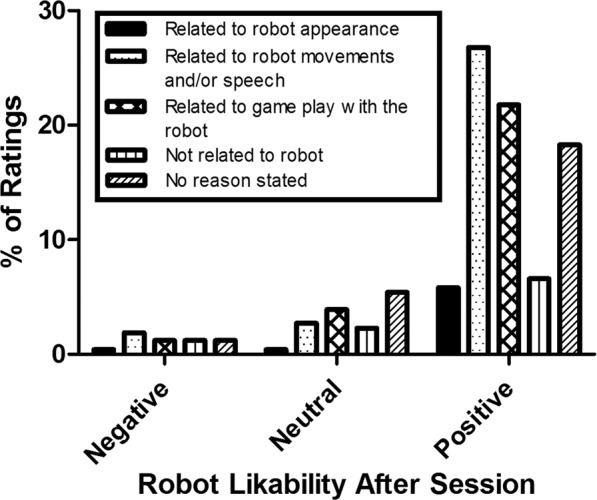
Reasons for robot likability ratings, displayed separately for negative, neutral and positive ratings.

Additionally, in 79.4% of the sessions, the child reported a positive robot likability score (see Fig. 3). The majority of reasons for a positive likability score were either related to the robot movements and/or speech (33.8%) or related to the game play with the robot (27.5%). Of the robot likability ratings, 14.8% was neutral and 5.8% was negative. The majority of reasons for neutral likability ratings were either related to the game scenario with the robot (26.3%) or no reason was stated (36.8%). Of the negative likability scores, 33.3% was related to movements and/or speech of the robot and 20.0% was related to the game play with the robot.

## Discussion

Our aim was to present an intervention protocol for children with ASD, who can highly benefit from the use of technology in their therapy, combining motivational components of both PRT and robotics. The current protocol is the first in designing specific game scenarios for a 20-week intervention based on motivational techniques of PRT, an established intervention for children with ASD. Moreover, with this protocol, we aimed to account for individual differences in target behaviour and interests among children with ASD by using nine different game scenarios with seven levels of complexity. Furthermore, as little is known about treatment adherence and acceptability of robot-assisted interventions for children with ASD, the current study adds to the state of the literature by exploring both child and parent ratings with reasons.

Overall, children with ASD in our study could adhere to the treatment protocol, showed positive affect after the robot-assisted therapy sessions and showed high robot likability scores. Although affect ratings after the session where slightly lower in the high ASD severity group compared with the moderate ASD severity group, these ratings were also mainly positive. Most reasons for positive affect ratings were categorized as related to the second part of the therapy session (i.e. without the robot), in which the child could play a self-chosen game with the parent, while the parent implemented PRT techniques. This finding is in line with an earlier study^15^ in which children with ASD reported the parts of the therapy without the robot (but with a human trainer) as more enjoyable compared with the parts including a robot. These findings suggest that it is important to combine specific motivational features of robotics with those of already established treatment models for ASD, as has been recommended earlier^17,23,25^. Yet, within PRT as an established treatment for children with ASD, the robot can be added when this is desirable for following the child’s interests.

Within our sample, positive robot likability ratings as measures for robot acceptability were obtained in most therapy sessions, with reasons related to the robotic movements, speech, and game scenarios. This may indicate that the child’s motivation can be enhanced when the interaction with the robot is meaningful to the child by using game scenarios. Indeed, a previous study^20^ found that vocalizations may increase more during an interactional task with a contingent robot (i.e. responding to the child’s behaviour) compared with a robot showing non-contingent reinforcement. However, not all robot likability ratings were positive and preschool-age children had lower likability ratings, which suggests that the game scenarios for robot-child interaction may fit less well with the interests of younger children with ASD.

In line with child ratings, parent ratings of acceptability of the robot were mainly positive, since parents noticed that the child was enthusiastic and motivated during the sessions and showed initiations at home and at school by talking to classmates about the robot. Parents of both girls and boys showed high ratings of robot value, although parents of girls showed a slightly higher score. Involvement of parents in the robot-assisted intervention is very important for observing child’s behaviour outside the sessions and facilitating generalization of learned skills^39^. The use of a robot within PRT may facilitate initiations in children with ASD within the natural environment, which are pivotal skills for further social-communicative development^28^. However, whether use of a robot within therapy facilitates initiations in children with ASD beyond the effects of PRT, is yet unclear and currently under study.

One main reason for lower parent’s ratings was a delay in robot’s responses. Indeed, some robotic responses may not be fast enough to assure contingent reinforcement^40^. Although we attempted to design the game scenarios to assure direct and natural reinforcement, there was a delay of a few seconds between (1) the therapist initiating the reinforcement by clicking a button and (2) the robot providing the reinforcement. Also, when the child showed an appropriate spontaneous initiation during the robot-assisted game scenario (e.g. “robot, can you stand now”?), these could only be reinforced when specific movements were pre-programmed. Since direct reinforcement is a key PRT motivational technique, this issue limits the use of a robot in established interventions for children with ASD. Furthermore, lower parental ratings of robot value were mainly due to the game scenarios being either too easy or too difficult for the child. Although we aimed to tailor the robot-assisted scenarios more to the specific needs of each child compared to what has earlier been done^15,26^, nine different game scenarios with seven different levels of complexity was insufficient for the needs of each individual child. Children with ASD tended to reject the robot-assisted game scenarios when noticing that the robot could not perform autonomously or when the scenarios became too predictable. In contrast, the game scenarios may have been too challenging for other children with ASD and the therapist could not easily amend the difficulty during the robot-assisted session. This advocates increasing the flexibility of robot behaviour by developing a larger library of game scenarios with a high diversity in complexity, which can be easily upgraded or downgraded by the therapist during the therapy session.

### Limitations

Although it was meaningful to explore child affect ratings, we did not compare this with a ‘PRT-only’ group. This may provide better insight in whether the effect of motivational components of PRT on child affect can be significantly enhanced by inclusion of a robot. Furthermore, the number of participants in some subgroups within the exploratory analyses was low which limits conclusions that can be drawn from this data. Also, the reliability of child’s self-reported ratings may have been limited, since children with ASD show impairments in recognizing their own mental states and intentions^41^. In our study, about only half of the robot likability scores were directly related to the robot in the preschool age group, while this was over 66% of ratings by school-aged children. This may reflect a predictive lack of understanding of one’s own mental state or preferences in preschool-aged group, or an underdeveloped ability to verbally express reasons for ratings. Yet, child affect and likability scales that were used in this study were highly simplified and visual information was combined with verbal categorical response options, which is preferable when asking children about likability or emotional states^42^. Although many preschool-aged children are able to provide reliable self-reported ratings when providing an age-appropriate scale^43,44^, validated self-report scales that can be used to assess young children with ASD’s affect and likability ratings during therapy are currently lacking and should be developed in the future. Moreover, we did not investigate how affect and likability differed over the course of the intervention. Since child outcomes of robot-child interaction might be affected by the number of trials^45^, it would be interesting in future research to investigate whether affect and likability ratings differ related to the duration of robot-assisted interventions.

### Recommendations

In summary, we recommend that the following points should be taken into account when developing robot-assisted interventions for children with ASD: (1) combine the use of a robot with motivational components of established treatment models for ASD while emphasizing parent-child interaction in the sessions besides robot-child interaction, (2) use game scenarios that fit well with child’s interest in each session, especially for younger children with ASD, (3) assure that the child can be directly (without delay) and naturally reinforced by minimizing the robot’s response delay and the time the therapist needs to switch between a pre-programmed scenario and text-to-speech module, and (4) increase flexibility of the robot to match needs of each individual child by using a larger library of game scenarios, that can be easily amended by the therapist during the session. When incorporating these components, we are closer to effectively design robot-assisted interventions that might be of benefit to a larger group of children with ASD. This study contributes to the increased efforts to develop new technologies for use in mental health care interventions for children with psychiatric disorders.

## Supplementary information

Supplementary Information

Supplementary Information2
