# Supplementary material for: Adherence and acceptability of a robot-assisted Pivotal Response Treatment protocol for children with autism spectrum disorder
Source: Sci Rep. 2020 May 15;10:8110. doi: 10.1038/s41598-020-65048-3 (PMC7229010; doi:10.1038/s41598-020-65048-3)
Supplement: Supplementary file 1 — Supplementary Information [file 41598_2020_65048_MOESM1_ESM.docx]

Supplementary Information

Adherence and acceptability of a robot-assisted Pivotal Response Treatment protocol for children with autism spectrum disorder

Iris van den Berk-Smeekens^1,2,^*

(1) Department of Cognitive Neuroscience, Donders Institute for Brain, Cognition and Behaviour, Radboud University Nijmegen Medical Centre, P.O. Box 9104, 6500 HB Nijmegen, The Netherlands

(2) Karakter Child and Adolescent Psychiatry University Centre, Reinier Postlaan 12, 6525 GC Nijmegen, The Netherlands

Martine van Dongen-Boomsma^1,2^

(1) Department of Cognitive Neuroscience, Donders Institute for Brain, Cognition and Behaviour, Radboud University Nijmegen Medical Centre, P.O. Box 9104, 6500 HB Nijmegen, The Netherlands

(2) Karakter Child and Adolescent Psychiatry University Centre, Reinier Postlaan 12, 6525 GC Nijmegen, The Netherlands

Manon W. P. De Korte^1,2^

(1) Department of Cognitive Neuroscience, Donders Institute for Brain, Cognition and Behaviour, Radboud University Nijmegen Medical Centre, P.O. Box 9104, 6500 HB Nijmegen, The Netherlands

(2) Karakter Child and Adolescent Psychiatry University Centre, Reinier Postlaan 12, 6525 GC Nijmegen, The Netherlands

Jenny C. Den Boer^3^

(3) Karakter Child and Adolescent Psychiatry, Postbus 68, 6710 BB Ede, The Netherlands

Iris J. Oosterling^2^

(2) Karakter Child and Adolescent Psychiatry University Centre, Reinier Postlaan 12, 6525 GC Nijmegen, The Netherlands

Nienke C. Peters-Scheffer^4,5^

(4) Behavioural Science Institute, Radboud University Nijmegen, PO Box 9104, 6500 HE Nijmegen, The Netherlands

(5) Driestroom, PO box 139, 6660 AC Elst, The Netherlands

Jan K. Buitelaar^1,2^

(1) Department of Cognitive Neuroscience, Donders Institute for Brain, Cognition and Behaviour, Radboud University Nijmegen Medical Centre, P.O. Box 9104, 6500 HB Nijmegen, The Netherlands

(2) Karakter Child and Adolescent Psychiatry University Centre, Reinier Postlaan 12, 6525 GC Nijmegen, The Netherlands

Emilia I. Barakova^6^

(6) Faculty of Industrial Design, University of Technology, Eindhoven, P.O. Box 513 5600 MB Eindhoven, The Netherlands

Tino Lourens^7^

(7) TiViPE, Kanaaldijk ZW 11, 5706 LD Helmond, The Netherlands

Wouter G. Staal^1,2,8^

(1) Department of Cognitive Neuroscience, Donders Institute for Brain, Cognition and Behaviour, Radboud University Nijmegen Medical Centre, P.O. Box 9104, 6500 HB Nijmegen, The Netherlands

(2) Karakter Child and Adolescent Psychiatry University Centre, Reinier Postlaan 12, 6525 GC Nijmegen, The Netherlands

(8) Institute for Brian and Cognition, Leiden University, P.O. Box 9600 (C2-S), 2300 RC Leiden, Netherlands

Jeffrey C. Glennon^1^

(1) Department of Cognitive Neuroscience, Donders Institute for Brain, Cognition and Behaviour, Radboud University Nijmegen Medical Centre, P.O. Box 9104, 6500 HB Nijmegen, The Netherlands

**Correspondence**

Correspondence concerning this article should be addressed to Iris van den Berk-Smeekens*, Department of Cognitive Neuroscience, Donders Institute for Brain, Cognition and Behaviour, Radboud University Nijmegen Medical Center, and Karakter Child and Adolescent Psychiatry University Centre, Reinier Postlaan 12, 6525 GC Nijmegen, The Netherlands, telephone number: 003124-3512222, email address: i.smeekens@karakter.com.

**Acknowledgements**

This study was funded by a ZonMW Grant (Program Translational Research, project number 95103010) and by Karakter, expert center for the child- and adolescent psychiatry in The Netherlands. The study was further supported by the EU-AIMS (European Autism Interventions) programme which received support from Innovative Medicines Initiative Joint Undertaking Grant No. 115300, the resources of which are composed of financial contributions from the European Union’s FP7 Programme, and from the European Federation of Pharmaceutical Industries and Associations (EFPIA) companies’ in-kind contributions. This study was part of a larger randomised clinical trial to the effectiveness of PRT for children with ASD (PicASSo project, registered at 01/08/2014 at the Netherlands Trial Register; https://www.trialregister.nl/trial/4487; NL4487/NTR4712).

The authors thank all the children, parents and teachers that participated in the study, as well as the health care professionals of Karakter, interns and research assistants for their time and commitment which made the study possible.

**Supplementary Information**

**Type of robot**

The NAO robot (see Figure S1) was used in the robot-assisted PRT. Currently, the NAO robot is the most widely used robot in treatment for children with ASD^21,23^. The NAO robot is a humanoid robot, developed by Aldebaran Robotics. The NAO robot is 58 cm in height and includes 25 degrees of freedom (i.e. motors to control the head, arms, hands and legs of the robot and LEDs to control the lights in the eyes and ears). With these joints, the robot can be programmed to perform human-like movements, such as cheering, shaking a hand, nodding and eye-blinking. Additionally, the speech of the NAO robot is produced by a speech synthesizer and played through speakers that are integrated in the NAO robot. The NAO robot was used in the current study for several reasons: 1) the appearance of the NAO robot is engaging for children with ASD^40^, 2) it has highly simplified facial and bodily features, which may lower the possibility of overstimulation in these children^24^, 3) the NAO robot is a humanoid robot, which has the greatest potential of generalization of learned skills outside the robot-assisted therapy context^5^, and 4) NAO was used in several previous studies evaluating effects of robot-assisted therapy in ASD on similar social-communicational target behaviors as those that we aim to target in our larger study^13,15,16,46^.


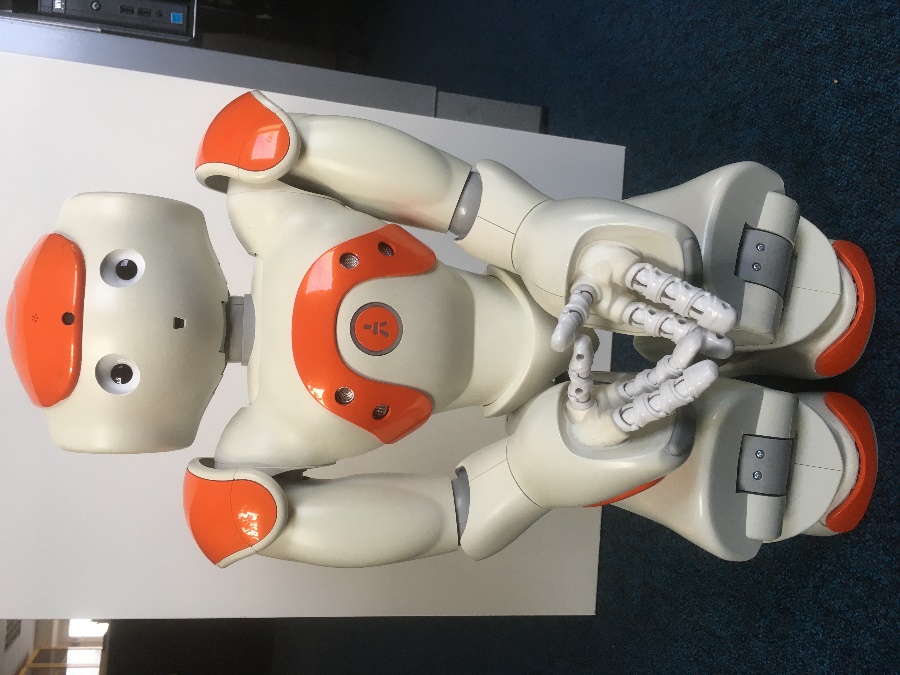


**Fig. S1** NAO robot that is used within robot-assisted PRT

**Controlling robot behavior**

In this study we chose to use the NAO robot in a tele-operated mode, meaning that the therapist is (remotely) controlling the robot and that the robot had no autonomous behavior, as this is done in most studies incorporating robots in children with ASD^23^. The advantage of this tele-operated mode is that the therapist is able to sensitively read how a child acts and responses in the therapy, and so can control the robot accordingly to provide an appropriate action or response^23^. To control the NAO robot, the visual programming environment TiViPE^47^ was used. With this program, pre-programmed game scenarios have been developed through co-creation between therapists trained in PRT and robotics specialists. In addition to the pre-programmed scenarios, a text-to-speech module was developed that could be activated when prompting or reinforcement was needed or the pre-programmed scenario did not match the child's response in a specific situation. The therapist could switch between the pre-programmed scenario and the text-to-speech module at any time during the therapy session (see flow diagram in Figure S2). The pre-programmed parts of the robot scenario included : 1) a state that controlled the speech and/or movements of the robot (rectangle-shaped boxes), and 2) a state that allowed the therapist to select the robot behavior that was in accordance with the child's behavior by pressing a key "y" (yes) or "n" (no) (diamond-shaped boxes).

To increase the predictability of robot behavior, only a small sample of movements was used, including 1) providing a hand and shaking the child's hand when saying "hello" and "bye" 2) blinking and/or cheering when the child showed appropriate behavior, 3) raising arms slightly when asking a question, 4) nodding when responding with "yes" and shaking head when responding with "no", and 5) behaviors that were functionally related to the game scenario, such as placing a hand on the box, making a sliding movement when opening the box, and pointing to a card. Additionally, the text files that provided input for the speech synthesizer where adjusted to make the robot speech as natural and understandable as possible. This required adding small time delays between words within one sentence (e.g. 'I_(*delay)*_have_more_animal_puzzles' rather than 'I_have_more_animal_puzzles') or to include some words in the text files using phonetic spelling rather than actual spelling (e.g. 'zebraas' rather than 'zebra's' in Dutch). As a pilot, typically developing children between 3-8 years old were asked to play different game scenarios and based on these observations, small adjustments were made in the text files prior to using the scenarios within the robot-assisted PRT for children with ASD.

**Fig. S2** Pre-programmed game scenario and text-to-speech module

**Game Scenario Development and Motivational Techniques of PRT**

To optimize the game scenarios for use within the robot-assisted PRT sessions for children with ASD, techniques of PRT are incorporated into the game scenario development. Within PRT, procedures are used that have been shown to increase the motivation for learning new tasks and behaviors in children with ASD, often referred to as motivational techniques^32,48^. These techniques will be described below in great detail.

**Technique 1: Child choice**

The motivation of children with ASD can be increased by the incorporation of child-chosen and preferred materials and activities into learning opportunities^32,49^. Since robots may be intrinsically appealing to many children with ASD^5^, we expected that using a robot during PRT relates to the preferences of these children and may increase their motivation. Additionally, robot scenarios have been developed with nine different games that are developmentally appropriate for children aged 3-8 years and were often used during PRT for young children with ASD in the outpatient treatment facility of Karakter: three different puzzle games (i.e. a puzzle with different animals, a puzzle with a magnetic boat, a puzzle with trucks), three different Lego© games (building a plane, a car, or a house for older children; building a plane, ducks or a house with Duplo© for younger children), and three different card ("Quartet") games (cars with Frog & Friends, cards with sea animals, cards with fairytale characters). The type of game (i.e. puzzles, Lego©, or cards) could be selected by the therapist before start of each therapy session, based on parental information on child's preferences during the first therapy sessions and based on child's choice during therapy in later sessions. Additionally, the kind of game (i.e. type of either puzzle, or Lego©, or cards) was chosen by the child during the interaction with the robot and the therapist could select the robot scenario based on the choice of the child. Also, if the child wanted to talk with the robot about a different subject during playing the game than that was pre-programmed in the game scenario, the text-to-speech module could be activated by the therapist and the robot could respond accordingly.

**Technique 2: Child attending and providing a clear opportunity to respond**

One of the main PRT techniques is providing a clear 'learning' opportunity for the child to respond, related to the child's interests^48^. Prior to presenting a learning opportunity, the therapist must have the child's interest and attention^28^. The child's attention is often drawn by placing a desired item where the child cannot obtain it, by breaking up preferred items or activities and by short time delays (i.e. waiting for a child to initiate, instead of providing objects or help immediately)^48^. Learning opportunities were included in the game scenarios by 1) placing the desired materials in a closed box before the game starts, 2) providing the child with only parts of the game materials at once by using a box that the robot could slide open, and 3) providing only parts of information about a game (e.g. robot says: 'I know how we can play this game' and waits for the child to initiate by asking the robot to explain how the game is being played). Learning opportunities were only provided if the child was interested in the robot and the game at that moment. If the child's attention was drawn to another game or subject, the therapist tried to use the text-to-speech module to respond accordingly. If the child lost attention to the robot during the game, the game scenario was aborted to maintain child's motivation for the therapy session.

**Technique 3: Interspersing maintenance tasks**

Another technique that has been shown to increase the motivation of children with ASD is to intersperse previously learned tasks (i.e. maintenance tasks) with new tasks (i.e. the target behavior in a specific therapy session) that the child has not acquired yet^32^. Interspersing learning opportunities for maintenance tasks with learning opportunities for new tasks often result in the child experiencing a high degree of success, which in turn, may lead to more attempts of the child^32^. For each child, target behavior was defined based on information of parents and the coordinating therapist before start of the PRT. Additionally, during the first PRT session, a 15-minute baseline session was administered measuring levels of spontaneity on different behaviors that are targeted by PRT (e.g. 2-or 3-word sentences, the use of multiple cues, asking for an object/activity, requesting help, protesting, wh-question asking (i.e. questions of what?, where?, who's? which?), and conversational skills such as asking through, making statements and initiating a conversation). Based on this information, a game scenario was selected for each child that included learning opportunities for both maintained (easy) and new (difficult) tasks. Since target behavior and interests in game materials could differ highly between children with ASD, 9 different therapeutic game scenarios were created, each with 7 different levels of complexity. The game scenario and level of complexity could be selected by the therapist before the robot-assisted PRT session was initiated. Table 4 provides an overview of the levels of complexity of the pre-programmed game scenarios.

Besides combining different target behaviors, interspersing maintenance and new tasks was also realized by adjusting the level of prompting (i.e. the help that the child received for showing appropriate behavior) throughout the robot-assisted game scenario. With the text-to-speech module, the therapist could provide the child with 4 levels of prompting: 1) wait prompt (i.e. waiting for the child to respond), 2) open question prompt (i.e. "what can you ask me now?"), 3) fill-in prompt (i.e., "you can ask me: "what is...""), and 4) tell prompt (i.e. "you can ask me: "what is inside the box?""). For each child, the level of prompting could be interspersed between easy (e.g. use of tell prompt as a maintenance task) and difficult (e.g. use of wait prompt as a new task) during the robot-assisted part of the session. Parts of the prompts were pre-programmed in the text-to-speech module and could be adjusted by the therapist to match the target behavior of the child in each individual situation.

Table S1.

| Complexity level | Target behavior |
| --- | --- |
| 1 | 2- or 3-word sentences, multiple cues |
| 2 | 2- or 3-word sentences, multiple cues, asking for object/activity |
| 3 | asking for object/activity, requesting help, protesting |
| 4 | requesting help, protesting, questions of what?, where?, who's? which? |
| 5 | protesting, questions of what?, where?, who's? which? |
| 6 | questions of what?, where?, who's? which?, supplementary questions |
| 7 | supplementary questions, making statements, initiating conversation |

**Technique 4: Direct and natural reinforcement**

With the use of direct and natural reinforcement, children with ASD learn that appropriate initiatives are tied to a consequence, which makes communication functional^48^. Direct reinforcement refers to immediately providing the reinforcer contingent upon the child's behavior^48^. Natural reinforcers are directly and functionally related to a task (e.g. providing a ball when a child asks "can I have the ball?") and have shown to better facilitate learning in children with ASD compared to arbitrary reinforcers that are unrelated to a task (e.g. providing candy when a child makes a puzzle)^32,50,51^. The game scenarios within the robot-assisted PRT are designed to provide a direct and natural reinforcement upon the child's behavior. For instance, when the child asks: "robot, can you open de box?" the therapist directly controls the robot in opening the box by pressing the appropriate key. Also, when a child takes an initiative that was not anticipated in the pre-programmed game scenario, the therapist could use the text-to-speech module to provide a direct and natural reinforcement.

**Technique 5: Reinforcement of attempts**

Reinforcing appropriate attempts for showing the target behavior has shown to increase the responsiveness during teaching interactions in children with ASD^32,48,52^. However, only attempts that are free of self-stimulatory or disruptive behavior should be rewarded^47^. In the programming environment that was used to control the robot, the therapist pressed "y" (yes) within the game scenario when the child showed the target behavior or an appropriate attempt, and a direct and natural reinforcement was provided by the robot. However, when the child did not initiate spontaneously or the attempt was deemed inappropriate, the therapist pressed "n" (no) and the text-to-speech module was activated to prompt the child in showing an appropriate attempt to the target behavior.

In Box 1, an example of a learning opportunity for the robot-assisted PRT is provided.

Box S1.

| Mark has chosen the lego game, building a lego helicopter in play with the robot. He starts with a few lego blocks, with which he can build the first part of the helicopter. When he has finished with the blocks, he looks at the robot, but doesn’t initiate a question. The PRT therapist controls the robot in providing an open question prompt: “what can you ask me now?”. Then, Mark shows an initiation: “Can I have block?”. The PRT therapist immediately controls the robot in providing a natural reward for the appropriate initiation attempt: the robot slides the box containing more blocks further open and says: “you can take more blocks from the box”. Mark takes the blocks and continues building the helicopter. |
| --- |

**Additional references Supplementary Information**

46. Kaboski, J. R. *et al.* Brief report: a pilot summer robotics camp to reduce social anxiety and improve social/vocational skills in adolescents with ASD. *J. Autism Dev. Disord.* **45,** 3862-3869 <https://doi.org/10.1007/s10803-014-2153-3> (2015).

47. Lourens, T. & Barakova, E. I. User-friendly robot environment for creation of social scenarios. *Lect. Notes*

*Comput. Sc. LNCS6686,* 212-22 (2011).

48. Koegel, R. L. & Koegel, L. K. *Pivotal Response Treatments for autism: communication, social and academic development* (Paul H. Brookes Publishing Co, Baltimore, 2006).

49. Koegel, R. L., Dyer, K. & Bell, L. K. The influence of child‐preferred activities on autistic children's social behavior. *J. Appl. Behav. Anal.* **20,** 243-252 <https://doi.org/10.1901/jaba.1987.20-243> (1987).

50. Koegel, L. K. & Koegel, R. L. Motivating communication in children with autism in *Learning and cognition*

*in autism* (eds. Schopler, E. & Mesibov, G. B.) 73-87 (Springer, New York, 1995).

51. Koegel, R. L. & Williams, J. A. Direct versus indirect response-reinforcer relationships in teaching autistic

children. *J. Abnorm. Child Psych.* **8,** 537-547, <https://doi.org/10.1007/BF00916505> (1980).

52. Koegel, R. L., O'Dell, M. & Dunlap, G. Producing speech use in nonverbal autistic children by reinforcing

attempts. *J. Autism Dev. Disord.* **18,** 525-538 <https://doi.org/10.1007/BF02211871> (1988).

**Table legends**

**Table S1.** Levels of Complexity of Game Scenarios for Robot-Assisted PRT

**Box S1.** Robot-Assisted Learning Opportunity
