# Supplementary material for: Adherence and acceptability of a robot-assisted Pivotal Response Treatment protocol for children with autism spectrum disorder
Source: Sci Rep. 2020 May 15;10:8110. doi: 10.1038/s41598-020-65048-3 (PMC7229010; doi:10.1038/s41598-020-65048-3)
Supplement: Supplementary file 2 — Supplementary Information2 [file 41598_2020_65048_MOESM2_ESM.pdf]

RESEARCH PROTOCOL

Title: ‘An exploratory study on the effectiveness of Pivotal Response Treatment and a robot-based Pivotal Response Treatment on social and communicative skills in children with autism spectrum disorder’

PROTOCOL TITLE

'An exploratory study on the effectiveness of Pivotal Response Treatment and a robot-based Pivotal Response Treatment on social and communicative skills in children with autism spectrum disorder'

**Protocol ID**	NL50509.091.14<br>(CMO nr. 2014-1263)
**Short title**	PicASSo (short for Prt based Intervention with robots for training Children with AutiSm in SOcial and communicative skills)
**EudraCT number**	N/A, NTR nr. 4712
**Version**	5
**Date**	02-04-2015
**Coordinating investigator/project leader**	J. C. (Jeffrey) Glennon, PhD<br>Assistant professor<br>Radboud University Nijmegen Medical Centre<br>Department of Cognitive Neuroscience<br>Postbox 9101<br>6500HB Nijmegen<br>The Netherlands<br>024-3610362<br>Email: Jeffrey.glennon@radboudumc.nl
**Principal investigator(s) (in Dutch: hoofdonderzoeker/ uitvoerder)**	J. K. (Jan) Buitelaar, MD PhD<br>Professor<br>Radboud University Nijmegen Medical Centre<br>Department of Cognitive Neuroscience<br>Karakter centre for child and adolescent psychiatry<br>024-3610655<br>Email: Jan.Buitelaar@radboudumc.nl<br><br>W. G. (Wouter) Staal, MD PhD<br>Child and adolescent psychiatrist<br>Karakter centre for child and adolescent psychiatry

	Nijmegen<br>024-3512222<br>Email: w.staal@karakter.com<br><br>J. C. (Jenny) den Boer, MD<br>Child and adolescent psychiatrist<br>Karakter centre for child and adolescent psychiatry<br>Ede<br>0318-676611<br>Email: j.denboer@karakter.com<br><br>M. (Martine) van Dongen-Boomsma, MD PhD<br>Child and adolescent psychiatrist<br>Karakter centre for child and adolescent psychiatry<br>Nijmegen<br>024-3512222<br>Email: m.vandongen-boomsma@karakter.com<br><br>E. I. (Emilia) Barakova, PhD<br>Assistant professor<br>Technical University Eindhoven<br>Department of Industrial Design<br>040-2473563<br>Email: e.i.barakova@tue.nl
**Sponsor (in Dutch: verrichter/opdrachtgever)**	J. K. (Jan) Buitelaar, MD PhD<br>Professor<br>Radboud University Nijmegen Medical Centre<br>Department of Cognitive Neuroscience<br>Karakter centre for child and adolescent psychiatry<br>024-3610655<br>Email: Jan.Buitelaar@radboudumc.nl
**Subsidising party**	ZonMW – The Netherlands Organisation for Health Research and Development<br>Postbox 93245<br>2509 AE The Haque, The Netherlands

	Contact person: Janine Blom<br>Programme Officer team Science & Innovation<br>070-3495137<br>Email: jblom@zonmw.nl
**Independent expert**	P. C. M. (Pierre) Herpers, MD<br>Child and adolescent psychiatrist<br>Vluchtheuvellaan 6<br>8871 DN Zetten<br>Karakter centre for child and adolescent psychiatry<br>0488-469611<br>Email: p.herpers@karakter.com
**Laboratory sites <*if applicable*>**	N/A
**Pharmacy <*if applicable*>**	N/A

PROTOCOL SIGNATURE SHEET

**Name**	**Signature**	**Date**
**Head of Department:**<br>G. (Guillén) Fernández, MD PhD<br>Professor<br>Radboud University Nijmegen Medical Centre<br>Department of Cognitive Neuroscience		
**Principal Investigator:**<br>J. K. (Jan) Buitelaar, MD PhD<br>Professor<br>Radboud University Nijmegen Medical Centre<br>Department of Cognitive Neuroscience		

TABLE OF CONTENTS

1. INTRODUCTION AND RATIONALE 11
2. OBJECTIVES 15
3. STUDY DESIGN 17
4. STUDY POPULATION 19
4.1 Population (base) 19
4.2 Inclusion criteria 19
4.3 Exclusion criteria 19
4.4 Sample size calculation: N/A 20
5. TREATMENT OF SUBJECTS 21
5.1 Investigational product/treatment 21
5.2 Use of co-intervention (if applicable) 24
5.3 Escape medication (if applicable) 24
6. INVESTIGATIONAL PRODUCT: N/A 25
7. NON-INVESTIGATIONAL PRODUCT: N/A 25
8. METHODS 26
8.1 Study parameters/endpoints 26
8.1.1 Main study parameter/endpoint 26
8.1.2 Secondary study parameters/endpoints (if applicable) 27
8.1.3 Other study parameters (if applicable) 28
8.2 Randomisation, blinding and treatment allocation 30
8.3 Study procedures 30
8.4 Withdrawal of individual subjects 33
8.4.1 Specific criteria for withdrawal (if applicable) 33
8.5 Replacement of individual subjects after withdrawal 33
8.6 Follow-up of subjects withdrawn from treatment 33
8.7 Premature termination of the study 33
9. SAFETY REPORTING 34
9.1 Section 10 WMO event 34
9.2 AEs, SAEs and SUSARs 34
9.2.1 Adverse events (AEs) 34
9.2.2 Serious adverse events (SAEs) 34
9.3 Annual safety report: N/A 35
9.4 Follow-up of adverse events 35
9.5 [Data Safety Monitoring Board (DSMB) / Safety Committee] 35
10. STATISTICAL ANALYSIS 36
10.1 Primary study parameter(s) 36
10.2 Secondary study parameter(s) 36
10.3 Other study parameters 37
10.4 Interim analysis (if applicable): N/A 38
11. ETHICAL CONSIDERATIONS 39
11.1 Regulation statement 39

11.2 Recruitment and consent....39
11.3 Objection by minors or incapacitated subjects (if applicable)....39
11.4 Benefits and risks assessment, group relatedness....40
11.5 Compensation for injury....40
11.6 Incentives (if applicable)....40
12. ADMINISTRATIVE ASPECTS, MONITORING AND PUBLICATION....41
12.1 Handling and storage of data and documents....41
12.2 Monitoring and Quality Assurance....41
12.3 Amendments....41
12.4 Annual progress report....41
12.5 End of study report....42
12.6 Public disclosure and publication policy....42
13. STRUCTURED RISK ANALYSIS: N/A....43
14. REFERENCES....43
Attachment 1....46
Attachment 2....47
Attachment 3....48

LIST OF ABBREVIATIONS AND RELEVANT DEFINITIONS

**APA**	**American Psychiatric Association**
**ASD**	**Autism Spectrum Disorder**
**AE**	**Adverse Event**
**AR**	**Adverse Reaction**
**CCMO**	**Central Committee on Research Involving Human Subjects; in Dutch: Centrale Commissie Mensgebonden Onderzoek**
**DSMB**	**Data Safety Monitoring Board**
**GCP**	**Good Clinical Practice**
**IC**	**Informed Consent**
**IQ**	**Intelligence Quotient**
**METC**	**Medical research ethics committee (MREC); in Dutch: medisch ethische toetsing commissie (METC)**
**PRT**	**Pivotal Response Treatment**
**(S)AE**	**(Serious) Adverse Event**
**Sponsor**	**The sponsor is the party that commissions the organisation or performance of the research, for example a pharmaceutical company, academic hospital, scientific organisation or investigator. A party that provides funding for a study but does not commission it is not regarded as the sponsor, but referred to as a subsidising party.**
**WBP**	**Personal Data Protection Act (in Dutch: Wet Bescherming Persoonsgevens)**
**WHO**	**World Health Association**
**WMO**	**Medical Research Involving Human Subjects Act (in Dutch: Wet Medisch-wetenschappelijk Onderzoek met Mensen**

SUMMARY

Rationale: Earlier studies focussing on the effectiveness of PRT and the effects of implementing robotics into the treatment of children with ASD show promising results but are limited due to methodological problems. The current study addresses these problems by conducting an exploratory cluster-randomized clinical trial to the effectiveness of PRT and PRT with the implementation of a humanoid robot.

Objective: The main objective is to investigate the effectiveness of robot-based PRT and PRT by a human trainer to care-as-usual in promoting social and communicative skills in the natural environment of the child and in improving mental health significantly. Secondary objectives are focused on the investigation of improvements in skills during the treatment and qualitative reports reflecting the usefulness of implementing a robot in the treatment of children with ASD. Also, physical markers of stress and social behaviour will be related to questionnaire data and qualitative reports.

Study design: The study includes a randomized (phase IIa – like) open three-group parallel clinical trial.

Study population: The target population of the exploratory study consists of 75 children diagnosed with ASD (*n* = 25 for each intervention group), aged 3-8 year without an intellectual disability (IQ > 70).

Intervention:
Subjects are cluster-randomly assigned to three intervention conditions:
1) Robot -based PRT on top of care-as-usual (Robot-based PRT condition)
2) PRT by a human trainer, on top of care-as-usual (PRT condition)
3) Care-as-usual

Main study parameters/endpoints:
1) Clinically significant response (reduction of more than 25%) on the SRS: improvement in social and communicative skills in the child's natural environment
2) Clinically significant response on the Clinical Global Impression – Improvement (CGI-I), measuring improvement in mental health (score much improved or very much improved).
3) Significant decrease on the ADOS severity score: decrease in ASD symptoms

Nature and extent of the burden and risks associated with participation, benefit and group relatedness:

Patients assigned to the three conditions are all expected to benefit from their treatment. Patients in the robot-based PRT condition and PRT condition receive care-as-usual (psycho-education and medical management) in addition to the intervention. Risks associated with participating in the study are estimated to be very low.

1. INTRODUCTION AND RATIONALE

Autism spectrum disorder (ASD) is characterized by persistent deficits in social communication and social interaction across multiple contexts and restricted, repetitive patterns of behaviour, interests, or activities (APA, 2013). Specifically, children with ASD show difficulties in a variety of social skills including turn-taking, sharing, assisting others, requesting information from others, introducing one self and responding to the behaviours of others (Macintosh & Dissanayake, 2006). These social difficulties can lead to serious problems when children enter school settings which include a more complex and demanding social environment (Fabes, Martin, & Hanish, 2009). School-aged children with autism were found to have smaller social networks, less reciprocated friendships and a lower quality of friendships (Kasari, Locke, Gulsrud, & Rotheram-Fuller, 2011). Also, in children with ASD, a lower quality of life is reported compared to their typically developing peers and this difference is consistent across lifespan (van Heijst & Geurts, 2014). Additionally, long-term outcomes for individuals with ASD are poor (Billstedt, Gillberg, & Gillberg, 2005) and due to the chronic course, heavy burden is placed on individuals with ASD and their families. Only a small minority of individuals with ASD is able to live independently, find a job and develop meaningful relationships throughout life (Howlin, Goode, Hutton, & Rutter, 2004). Consequently, the majority of individuals with ASD remains dependent on the support of parents, caregivers and professionals.

Due to the dependency on professional services of individuals with ASD and their families and due to a lack of income generation, the lifetime societal costs for an individual with ASD can raise to $3.2 million (Ganz, 2007). With an overall prevalence rate of ASD of around 1% of the population (Baird et al., 2006), the financial burden of ASD for the society is enormous. The high prevalence of ASD is also found in The Netherlands, which varies between 57 per 10.000 to 229 per 10.000 depending on the geographical region (Roelfsema et al., 2012). Prevalence rates of ASD continue to increase, which may reflect changes in diagnostic criteria, differences in methods across studies, or increased awareness and availability of services (Wing & Potter, 2002). Also, a true increase in prevalence cannot be ruled out, but the underlying causes are yet unknown (Rice et al., 2010).

Despite the high prevalence rates and costs of ASD, no effective pharmacologic interventions are currently available to treat the core symptoms of ASD. The available pharmacologic interventions are targeting comorbid symptoms in ASD, such as aggression, self-injurious behaviour, irritability, anxiety, hyperactivity or inattention (Myers & Johnson, 2007). In contrast, behavioural interventions based on applied behaviour analysis (ABA) have been proven effective to address problems in social

communication and reciprocal social interaction in children with ASD (Smith, McAdam, & Napolitano, 2007). Within ABA, intervention is based on the relationship between the child with ASD and the environment. That is, antecedents and consequences of social behaviour are determined and prompts (i.e. the help the child receives) are gradually faded (Lerman, Volkert, & LeBlanc, 2007). Approaches based on ABA principles often involve teaching discrete trials in a structured one-to-one teaching situation (Ghezzi, 2007). However, these approaches are highly labour intensive and costly and generalization of the learned skills to the natural environment of the child may not occur (Smith, 2001).

As a more naturalistic intervention procedure based on ABA principles, Pivotal Response Treatment (PRT) has emerged (Koegel, Koegel, Harrower, & Carter, 1999; Koegel & Koegel, 2006). Within PRT, the focus is on pivotal areas that, when targeted, lead to large collateral changes in other areas of functioning and responding (Koegel & Koegel, 2006). Pivotal areas that have been studied are motivation, responsivity to multiple cues, self-management and self-initiations (Koegel, Koegel, & McNerney, 2001). For instance, increasing motivation of children with ASD to respond by following child’s choice, natural reinforcement, rewarding attempts and task variation can lead to more learning opportunities and social interactions in the child’s natural environment (Koegel et al., 2001). Advantages of PRT are relatively small amount of intervention hours and the cost efficacy, because the focus in on pivotal areas targeted in the natural environment of children (Koegel et al., 1999). Also, generalization of the learned skills is often problematic in children with ASD and this is more likely to occur through PRT because of the naturalistic approach (Koegel & Koegel, 2006). Indeed, previous studies have shown that PRT is effective in the improvement of different social and communicative skills in children with ASD, including joint attention (Vismara & Lyons, 2007), turn taking (Harper, Symon, & Frea, 2008) asking questions (Koegel, Camarata, Valdez-Menchaca, & Koegel, 1998; Koegel, Koegel, Green-Hopkins, & Barnes, 2010) and spontaneous initiations (Kuhn, Bodkin, Devlin, & Doggett, 2008; Pierce & Schreibman, 1995).

Within the last decade, the possibility of using interactive environments within the treatment of children with ASD has received increased attention (Barakova, Gillessen, & Feijs, 2009; Dautenhahn & Werry, 2004; Diehl, Schmitt, Villano, & Crowell, 2012). Improvements in technology, especially regarding robotics, offer possibilities for the innovation of the treatment of children with ASD (Diehl et al., 2012). Technological applications such as robotics are intrinsically appealing to children with ASD and robots have been shown to generate a high degree of motivation to interact in these children (Scassellati, 2007). Additionally, children with ASD seem to be more responsive to

feedback provided though technology, which is less demanding for these children (Charlop-Christy, Le, & Freeman, 2000; Ozonoff, 1995). Also, because children with ASD have difficulty paying attention to the relevant cues that are needed in social interaction, they face difficulty in understanding other's behavior (Koegel et al., 1999). In contrast, robot behaviour can be controlled and easily adjusted in complexity to the individual needs of the child, which may lead to a safer and effective environment for interaction and learning (Gillesen, Barakova, Huskens, & Feijs, 2011). Indeed, earlier studies focused on implementing robots in the treatment of children with ASD showed promising results. For instance, Feil-Seifer and Matarić (2009) found that during interaction with a robot that is responsive to the child's behaviour, children with ASD showed an increase in speech directed to the robot and their parent. Also, Duquette, Michaud, and Mercier (2008) found that a group of low-functioning children with ASD showed increased shared focused attention when interacting with a robotic mediator. Additionally, in a recent study, Huskens, Verschuur, Gillessen, Didden, and Barakova (2013) showed that an ABA-based intervention with a humanoid robot is equally effective as an intervention with a human trainer in promoting self-initiated question asking in children with ASD.

Although the implementation of robots in the treatment of ASD seems promising, there is need for research focusing on generalization of learned skills into the natural environment of the child. Also, the pilot studies that have been conducted on this topic involve methodological limitations including small sample sizes (i.e. mostly 3 to 4 subjects), involving only qualitative reports of robot effects, the lack of diagnostic confirmation of ASD in subjects, and the lack of integration of robotics in an empirically supported treatment for ASD (Diehl et al., 2012; Scassellati, Admoni, & Matarić, 2012). Similarly, the majority of studies conducted to the effectiveness of PRT in children with ASD contains methodological limitations, including small sample sizes and the lack of an experimental design (Verschuur, Didden, Lang, Sigafoos, & Huskens, 2014).

The current study will address the limitations in earlier studies to both the effectiveness of implementing robots in the treatment of ASD and to the effectiveness of PRT by:

- conducting a exploratory cluster-randomized clinical trial, comparing 3 conditions of treatment with cluster randomization of subjects across conditions: 1) Robot-basedPRT + care as usual, 2) PRT (by a human trainer) + care-as-usual, and 3) care-as-usual;
-including 75 subjects with ASD in the clinical trial, 25 subjects in each condition;
-confirming diagnosis of ASD in all subjects by a structured instrument that serve as the gold standard for diagnostic confirmation in individuals with ASD;

-including both quantitative (i.e. standardized questionnaires and behavioural observation schedules) and qualitative (e.g. treatment fidelity, acceptance of robot in treatment) measures of treatment effectiveness;
-exploring possibilities of implementing a robot into treatment while using techniques of an empirically supported treatment for promoting social and communicative skills in children with ASD;
-measuring generalization of skills to the natural environment of the chid during and after the treatment and at 3 months follow-up.

Since early deficits in developmental processes in young children with ASD are related to poor outcomes later in life (Kasari, 2002) early intervention in ASD is important for improving quality of life and independence of individuals with ASD throughout their life. From early childhood to adolescence, children with ASD can make remarkable improvements and intervention during this period can provide long-lasting positive changes (Kasari, 2002). For an optimal use of this opportunity, it is important to determine which forms of treatment of ASD are effective an beneficial in young children with ASD. To draw valid conclusions regarding this issue, it is important to test the effectiveness of the intervention conditions for promoting social and communicative skills in a sample of young children with ASD. Therefore, the cluster-randomized clinical trial will be conducted in children diagnosed with ASD, aged 3-8 years with an intelligence quotient of >70.

2. OBJECTIVES

Primary Objective:
The main objective of the current study is assessing the effectiveness of an explorative robot-based PRT t on top of care-as-usual compared to care-as-usual only.
The primary hypothesis is:
1) Robot-based PRT is more effective compared to care-as-usual only in:
-promoting social and communicative skills in children with ASD (reduction of ASD symptoms within the natural environment of the child)
-providing a clinically significant improvement on mental health
-decreasing severity of ASD symptoms

Secondary objectives:
2) PRT provided by a human trainer is more effective compared to care-as-usual in:
-promoting social and communicative skills in children with ASD (reduction of ASD symptoms within the natural environment of the child)
-providing a clinically significant improvement on mental health
-decreasing severity of ASD symptoms

Besides measuring generalization of social and communicative skills into the natural environment of the child, the interest is in assessing the improvement in skills during the treatment and assessing the likability of the robot by the children with ASD and the affect of the child within the robot-based PRT. This provides more information on the usefulness of implementing a robot in the treatment of children with ASD.
3) Robot-based PRT and PRT provided by a human trainer are effective in:
-lowering the prompt level (i.e. help that is needed) for communicative skills in children with ASD during treatment
-heightening the number of learning moments the child shows spontaneous appropriate behaviour (e.g. initiations) on during the treatment
4) The robot that is used in the robot-based PRT shows a high likability by children with ASD
5) Children with ASD show positive affect during the robot-based PRT sessions

Also, child rearing pressure on parents is administered, because of the highly parent-focused PRT.
5) Robot-based PRT and PRT provided by a human trainer are both effective in decreasing the child rearing pressure experienced by parents.

Additionally, interest is in the relation between questionnaire data about social behaviour and qualitative reports on robot experiences, related to possible physical makers.
6) Qualitative affect scores during PRT are related to salivary cortisol levels
7) Social and communicative skills in children with ASD are related to salivary oxytocin and testosterone levels

STUDY DESIGN

The study is designed as a cluster-randomized (phase IIa – like) open three-group parallel clinical trial. Figure 1 (see attachment 1) provides an overview of the study procedure and time investment of subjects. After the selection of possible subjects by therapists, obtaining informed consent and administering initial measures for checking inclusion criteria (when recent information regarding this issues is not available in case files), subjects will be randomly assigned to one of three intervention conditions:

1) Robot-based PRT: Therapy is provided based on Pivotal Response Treatment with the implementation of a humanoid robot on top of care-as-usual, i.e. psycho-education and medical management (*n* = 25)

2) PRT: Pivotal Response Treatment is administered by a human trainer on top of care-as-usual, i.e. psycho-education and medical management (*n* = 25)

3) Care-as-usual: includes guidance of parents or intensive psychiatric family treatment, besides psycho-education of ASD and medical management (*n* = 25)

For 4 treatment locations of Karakter, subjects are randomized between robot-based PRT and care-as-usual, and for 3 treatment locations of Karakter, subjects are randomized between PRT and care-as-usual. Subjects are receiving one of the three intervention conditions, with a duration of 5 months. The robot-based PRT consist of 20 sessions of 45 minutes, with a frequency of one a week. Within care-as-usual, there are differences in hours of treatment between subjects. Therefore the number of treatment hours will be included as a covariate in the analyses (see section 9). A 3 month follow-up is included for all intervention conditions. The study will be conducted at different outpatient sites of Karakter centre for child and adolescent psychiatry, a specialized psychiatric hospital for treatment of children with complex psychiatric disorders such as ASD in the centre and eastern parts of the Netherlands. The interventions are administered under the supervision of a child psychiatrist or GZ-registered psychologist specialized in the treatment of children with ASD. The researcher that coordinates the study will be in contact with the responsible therapist at least once a week. Due to the content of the intervention conditions, both the subjects and the trainers (i.e. the professionals that provide the treatment) are aware of which intervention the subject is assigned to.

Regarding the robot-based PRTand the PRT condition, time investment for children for the parent-child treatment sessions is 630 min (10,5h). Total additional time investment for all the measures (initial, during treatment, evaluation and follow-up) for the children with ASD varies from 110-370 min (1,8 – 6,2h). For the parent or primary caregiver of the child, time investment for all the treatment sessions is 900 min (15h) and total additional time investment for measures is 140 min (2,3h) and for the teacher, time

investment is 60 min (1h). With regard to the care-as-usual condition, the additional time investment upon the regular time investment for treatment varies from 80 – 340 min (1,3 - 5,7h) for the child, is 140 min (2,3h) for the parent, and is 60 min (1h) for the teacher.

3. STUDY POPULATION

4.1 Population (base)

In this study, the focus is on children with ASD, aged 3-8 years, both males and females with an intelligence quotient of >70. Children are recruited on different outpatient treatment locations of Karakter. On all outpatient sites, parents from patients that are referred to Karakter for treatment in communication skills (after having received ASD diagnosis) are asked to participate in the study by their therapist or treatment coordinator in an appointment for discussing treatment advises. These parents will receive an information letter on the study. Parents are asked to fill in an informed consent form (see additional file E2) if they agree with participation of their child in the study.
A sufficient number of patients with ASD is available within Karakter to meet the target of 25 subjects for each intervention condition (i.e. 75 subjects in total).

4.2 Inclusion criteria

In order to be eligible to participate in this study, subjects must meet all of the following criteria:
-aged 3-8 years at start of the intervention.
-clinically diagnosed with ASD according to the *Diagnostic and Statistical Manual of Mental Disorders (DSM), fifth edition* (APA, 2013), *fourth edition of the DSM* (APA, 2000) or *International Classification of Diseases and Related Health Problems* (WHO, 1993). Diagnosis should be confirmed by the *Autism Diagnostic Observation Schedule* (ADOS) (De Bildt, Greaves-Lord, & De Jonge, 2013).
-a total intelligence quotient (TIQ) > 70.
-ability to speak with single words at minimum.
-at least one of the parents speaks Dutch to the child at home.

4.3 Exclusion criteria

A potential subject who meets the following criteria will be excluded from participation in this study:
-medication doses cannot be fixed during the study
-having received PRT earlier

4.4 Sample size calculation

N/A because of an exploratory study

5 TREATMENT OF SUBJECTS

5.1 Investigational product/treatment

The study consists of 3 intervention conditions, 1) Robot-based PRT on top of care as usual (robot-based PRT condition), 2) PRT by a human trainer on top of care-as-usual (PRT condition), and 3) care-as-usual.

Robot-based PRT condition

The robot-based PRT consists of 20 sessions, with the duration of 45 minutes. Within the robot-based PRT, the focus is on improving the social and communicative skills, regarding 1) *two (or more)-word verbalizations*, 2) *asking for an object/activity*, 3) *asking for help*, 4) *w-question asking*, 5) *protesting*, 6) *interrogating*, 7) *making a statement*, 8) *responding to multiple cues*. In each session, one or multiple learning goals are targeted. The content of the session depends on the learning goals of the specific child. During the session, focus is also on the *motivation* of the child, by e.g. following child's choice and alternating the content and difficulty of tasks. In accordance with the age of the child, learning moments are created approximately once a minute by playing games. Examples of games are playing with lego or duplo, wooden blocks, animal toys, quartet games, memory games and puzzles. Of the 20 sessions, 14 sessions involve the parent practicing with the child (see "PRT condition" for a detailed description of the different PRT sessions) and in these sessions, the first 15 minutes involves the child interacting with the robot. During the robot-child interaction, the parent learns the PRT techniques by observing the robot-child interaction, with explanation of the PRT therapist. After observing the robot-child interaction, the parents are instructed to practice the PRT techniques that they just observed. The robot is not involved in 7 PRT sessions, since these sessions are mainly focused on discussing the progress with parents.

Within 14 sessions, a humanoid robot called NAO from Aldebaran robotics is used (see Figure 2, attachment 2). The robot is 50cm tall, has 25 mechanical degrees of freedom, has digital cameras, speakers, microphones touch sensors and wireless communication capabilities. The robot as a "simple" face to prevent overstimulation in children with ASD. The robot can engage in interactive behaviour with the child, including speech, movements, and changing of face LEDs. The speech of the robot includes a pre-programmed Dutch female voice, since most of the human trainers are female and the aim is to simulate regular PRT sessions as much as possible. For the robot-based PRT sessions, pre-programmed scenarios with the inclusion of *learning moments* and *prompts* are created by a psychologist trained in PRT. Figure 3 provides an example of a pre-programmed scenario. The scenario has the form of a flowchart, which is used earlier in establishing treatments for individuals with ASD (Palmen, Didden, & Arts, 2008). A learning opportunity is

provided by the robot by presenting his hand. If the child gives a hand or says “hi”, the child shows behaviour that is appropriate in the situation and is rewarded with “thanks for greeting me” and provided by a hand shake (natural reinforcement). Attempts for showing appropriate behaviour are also rewarded, in accordance to principles of PRT (Koegel, 2014). If the child does not respond or shows behaviour that is inappropriate in the situation, a prompting sequence is initiated. A prompt is the level of help and guidance that is provided to the child and there are 4 levels included in the robot-based PRT, varying from least intensive to most intensive:
-*waiting prompt*: the robot waits 7 sec for the child to respond
-*open question prompt*: the robot asks: “what can you do now?”
-*fill in prompt:* the robot says: “fill in: ha..” while providing a hand
-*tell prompt:* the robot says: “please say hi (Dutch: “hallo”) to me and give me a hand”
Although the robot-based PRT is explorative, quality of the therapy is obtained by using evidence-based PRT techniques for ASD and by implementing sufficient learning moments into the treatment. A trained PRT therapist that is present during the treatment determines whether the child shows appropriate behaviour during the robot-based PRTsessions by pressing “y” (yes) or “n” (no). Also, when a *physical prompt* is required (i.e. performing an action together with the child) the therapist helps the child with this action while the robot waits. With the pre-programmed scenarios, it is easy to reassure that enough learning moments are created during the session and that all the prompting levels are included for each learning moment. In parallel to pre-programmed scenarios, a text-to-speech functionality is used during the sessions, which provides the ability for the therapist to instantly respond to initiations of the child and instantly provide reinforcement. The text-to-speech functionality is implemented to increase flexibility in following the child’s lead in the interaction.

A visual programming environment called TiViPe, short for Tino’s Visual Programming Environment (Lourens & Barakova, 2011) is used to program the scenarios with connecting modules and prompting sequences (see Figure 3, attachment 2). The flowcharts that are created by a PRT-trained psychologist are implemented into TiViPe. Also, this program is used by the therapist to execute the scenarios and provide instant feedback with the text-to-speech functionality by means of the robot. Within a earlier pilot study, TiViPe is used successfully to execute robot speech and movements within the treatment of children with ASD (Huskens et al., 2013).

The robot-based PRT is provided on top of care-as-usual. Before the start of the robot-based PRT, parents will receive 3 sessions of psycho-education. If applicable, medical management continues during the robot-based PRT.

PRT condition (human trainer)

The PRT condition includes PRT provided by a trained human PRT-therapist, as is the regular procedure for providing PRT within Karakter. The intervention includes 20 PRT sessions of 45 minutes. As in the robot-based PRT, the focus is on improving different social and communicative skills, depending on the learning goals of each child. The main focus is on teaching parents to implement PRT principles in social situations with their child, based on the strengths of parents.

Before the treatment starts, parents are informed about PRT and are provided information on the treatment procedure and deciding upon learning goals. In the first session, the PRT therapist observes the parent and child interacting. In session 2, the PRT therapist plays with the child and asks the parents to observe how PRT principles can be implemented. In the 3th session, the parents are asked how they think about implementing PRT principles and the PRT therapist informs the parents about her observations. Within sessions 4, 5, 7, 8, 10, 11, 13, 14, 16, 17 and 19, the parent practices PRT techniques while playing with the child and the PRT therapist functions as a coach for the parent and provides feedback. In sessions 6 and 15, the PRT therapist discusses with the parents whether progress is made in both the learning goals of the child and the implementation of the PRT techniques by the parents. In session 9, the progress is discussed with the parents and treatment coordinator and discussed is how to include the school or day care facility within the treatment. Within session 12, implementation of PRT within school or day care is further discussed and session 18 includes the practicing of teachers or day care attendants with PRT techniques. In the final (20th) session, the treatment is evaluated with the parents regarding progress of both the child and the parents. If further treatment is necessary, this is also discussed during the evaluation session.

Similar to the robot-based PRT condition, PRT is provided on top of care-as-usual including psycho-education and medical management (if applicable).

Care-as-usual

The care-as-usual includes the regular treatment for ASD that is provided in outpatient departments of Karakter. The care-as-usual condition includes psycho-education of ASD and medical management, supplemented by guidance of parents and other primary caregivers or intensive psychiatric family treatment.

5.2 Use of co-intervention (if applicable)

When subjects are assigned to the robot-based PRTcondition or PRT condition, care-as-usual is also provided, including psycho-education and the use of medication, if applicable to

the specific child. However, in these intervention conditions, dosages of medication should be fixed to obtain minimal interference with possible effects of the robot-based PRT or PRT.

5.3 Escape medication (if applicable)

It is not expected that escape medication for acute attacks, pain or other complains is needed during or due to the intervention conditions. However, when possible comorbid conditions may cause additional physical or mental complaints during the course of the study, medication is provided by and in consultation with a child psychiatrist that is responsible for the medical management protocol of the individual patient.

6. INVESTIGATIONAL PRODUCT N/A

7. NON-INVESTIGATIONAL PRODUCT N/A

8. METHODS

8.1 Study parameters/endpoints

8.1.1. Main study parameter/endpoint

-*Generalized social and communicative skills*

The main study parameter is social and communicative skills that the child shows in his/her natural environment, i.e. the generalization of skills. This is assessed by the Social Responsiveness Scale (SRS) (Roeyers, Thys, Druart, De Schryver, & Schittekatte, 2011). The SRS (child version and preschool version) is a 65-item questionnaire that is completed by a parent/primary caregiver and teacher/attendant of the child and is completed in approximately 15 minutes. The SRS inquires the child's ability to engage in reciprocal social behaviour in natural social settings, among other domains of autistic symptoms. The SRS consist of 5 subscales, i.e. *Social Awareness, Social Cognition, Social Communication, Social Motivation and Autistic Mannerisms.* Items include statements about the child's reciprocal social behaviour and are rated on a 4-point scale (*never true – almost always true*). A clinical responder on the SRS is defined as a reduction in score of more than 25%.

-Change of patient's illness

Another main outcome of the study is the change in illness during and after intervention. This is assessed by a blinded child psychiatrist by observing videos of the child interacting with a parent or another person (max. 15 minutes, not during a treatment session) with the Clinical Global Impression-Improvement (CGI-I) scale (Guy, 1976). The CGI-I is a 7-point scale (*very much improved – very much worse*) on which the psychiatrist rates how much the child's illness has improved or worsened during the intervention compared to the baseline state. A clinical responder is defined as being "much improved" or "very much improved" on the Improvement scale.

-Severity of ASD symptoms

Severity of ASD symptoms is assessed by the Autism Diagnostic Observation Schedule (ADOS-2) (De Bildt et al., 2013). The ADOS is administered by observing the child during a semi-structured observation schedule. With the ADOS, the clinician elicits social, communicative, stereotyped and play behaviour to observe possible symptoms of ASD. Activities are performed with a 40- to 60 minute protocol and different protocols exist for children with different verbal abilities. Observations of the clinician are categorized and a score is assigned for each domain of ASD

symptoms. A severity score is calculated based on Gotham, Pickles, and Lord (2009) and a change score is computed comparing the ADOS score at evaluation (after session 20) and at baseline.

8.1.2. Secondary study parameters/endpoints (if applicable)

-Communicative skills during PRT interventions

Specific communicative skills that are targeted by the PRT with the level of help (prompts) that is needed by the child to perform these skills are assessed in the robot-based PRT condition and PRT condition. In line with recent PRT guidelines (Koegel, 2014) a 15-minute PRT screening scenario is developed and simulated at 4 measurement points. For the 2nd (session 10) and 3rd (session 20, evaluation) measurements, the screening scenario involves the first 15 minutes of the PRT treatment session. In this scenario, the prompt level that the child needs for performing *two (or more)-word verbalizations*, *asking for an object/activity*, *asking for help*, *w-question asking*, *protesting*, *interrogating*, *making a statement* and *responding to multiple cues* is assessed. Levels of prompt include (from lowest to highest): spontaneously (i.e. no prompt), wait prompt, open question prompt, fill-in prompt, tell prompt, physical prompt and no response after physical prompt. The child is provided 3 opportunities to show the communicative skill within the screening scenario. The design of different screening scenarios is similar (i.e. 3 opportunities for each skill with the same sequence), but the playing materials that are used differ in each scenario. Scenarios are designed in a way that they can be provided by the robot (in the robot-based PRT condition) and by a human trainer (in the PRT condition).

With a PRT observation schedule that is used during regular PRT treatment within Karakter, the prompt level for each opportunity is scored by a trained rater by observing videos of the PRT screening sessions. For 20% of the subjects in the PRT conditions, the sessions are scored by a second rater to obtain a measure for interrater agreement.

-Spontaneous appropriate behaviour in learning moments during PRT

For 3 treatment sessions (session 1, 10, and 20) in the robot-based PRT condition and PRT condition, a percentage is calculated for the amount of learning moments the child shows spontaneous appropriate behaviour in. For each session, the number of spontaneous appropriate behaviours is divided by the total number of opportunities for showing spontaneous appropriate behaviour (i.e. learning moments

in that session). With comparing the percentages across the 3 sessions, progress in e.g. spontaneous initiations within the treatment is estimated.
As for the communicative skills during PRT, presence of spontaneous appropriate behaviour is scored by a +/- for each learning moment by raters that are trained in recognizing appropriate behaviour. Sessions for 20% of the subjects are scored a by a second rater to estimate inter-rater agreement.

-child rearing pressure on parents
As an additional secondary outcome, the child rearing pressure that is experienced by parents will be measured by the Dutch “Opvoedingsbelasting vragenlijst” (OBVL). The OBVL is a digitalized questionnaire that is administered in one of the parents in approximately 10 minutes. The OBVL contains 34 items and 5 scales: *Problems in Caregiver-Child Relation, Problems in Rearing, Depressive Mood, Role-restriction,* and *Health Complaints*. On a 4-points scale, parents respond to the degree on which the given statements are applicable to them.

-likability of the robot by the child
As a qualitative measure of the usefulness of implementing a humanoid robot into treatment of children with ASD, the likability of the robot by the child is assessed using a Visual Analogue Scale (VAS). After each robot-based PRT session (that involves interaction with the robot), the child indicates how much he or she likes the robot at that moment. The VAS include 5 points (scoring 4-0), including “I like the robot very much” (big thump up), “I like the robot” (small thump up), “I like the robot a little” (thump half way), “I dislike the robot” (small thump down), “I dislike the robot very much” (big thump down). For the likability of the robot, a mean score and a change score is computed.

-child’s affect during robot-based PRT sessions
Child’s affect is an important parameter for determining the acceptance and motivation of the child during the robot-based PRT sessions. Child’s affect is estimated by presenting the child with a VAS before and after each robot-based PRT session (that involves interaction with the robot). Children are asked to indicate how they feel at that moment on a 5-point scale, including “very happy” (big happy smilie), “happy” (small happy smilie), “a little bit happy” (neutral smilie), “not happy” (small sad smilie), and “not happy at all” (big sad smilie). Scores are ranging from 4-0 and for each session, a before and after score is estimated. From the before and

after scores of child's affect in each session a mean score and change score is computed.

-salivary cortisol, oxytocin and testosterone

In the two PRT conditions, salivary samples are collected for the assessment of cortisol, oxytocin and testosterone levels. Before and after session 1, 10 and 20, the child is asked to gently spit in a plastic tube. When spitting in a plastic tube is too difficult for the child, a cotton swab is used to collect the saliva. The investigator collecting the samples is instructed in patiently and carefully collecting saliva samples in young children. The parent/caregiver of the child has the option to decline the saliva collection in their child for any reason. Also, when the child shows resistance to the collection, this will be terminated. Parents of the children are asked to avoid that the child brushes his/her teeth during 1 hour prior to the saliva collection. For the detection of cortisol, oxytocin and testosterone, 3 ml of saliva is sufficient. Samples are transferred for storage in a -80°C freezer in the molecular laboratory from the RadboudUMC at the end of the testing day to preserve the hormones. Samples are coded with the participant number and number of collection moment (i.e. 01-01, 01-02 ....., 50-06). On the samples, the date and time of collection is written. The time of collection will be catalogued to minimize differences in hormone concentration between participants due to day/night rhythm. Samples will be analysed in the molecular laboratory of Dr. Jeffrey Glennon, Dept. of Cognitive Neuroscience, RadboudUMC. Cortisol, oxytocin and testosterone will be measured using competitive enzyme-based immunoassays using commercially available kits. The cortisol EIA typically displays an IC50 (50% B/B0) of approximately 180 pg/ml and a detection limit (80% B/B0) of approximately 35 pg/ml. The oxytocin EIA typically displays an IC50 (50% B/B0) of approximately 80 pg/ml and a detection limit (80% B/B0) of approximately 18 pg/ml. The testosterone EIA has a limit of detection of 80% B/B0: 6 pg/ml and a sensitivity: 50% B/B0 of 32 pg/ml. After analyzing the samples, these will be transferred to the Radboud Biobank and stored for a time period of 15 years.

8.1.3. Other study parameters (if applicable)

- Intelligence Quotient

When no recent information is available on IQ within the case file of the patient (i.e. an IQ measure that is administered within two years before start of the baseline) an initial measure for IQ is administered as an inclusion criterion (TIQ > 70).

Additionally, IQ will be included as a covariate, as this may intervene with the treatment outcomes when the mean IQ differs for the three treatment groups. For estimating TIQ the Wechsler Intelligence Scale for Children (WISC) (Kort et al., 2005), Wechsler Preschool and Primary Scale of Intelligence (WPPSI) (Hendriksen & Hurks, 2009) or Mullen Scales of Early Learning (MSEL) (Mullen, 1995) are administered, depending on the age of the child and appropriateness for the child.

-Age

Since earlier studies to the implementation of robots in the treatment of children with ASD do not report age effects, there is a need for assessing treatment effects in children with different ages. In the current study, age is included as a covariate in the analyses to assess whether implementation of a robot in the treatment is effective for children with different ages (e.g. preschool-aged children and school-aged children). Age of subjects at baseline is determined by analyzing case files.

-PRT fidelity of treatment implementation

Within PRT, it is important to assess whether clinicians are correctly implementing the specific PRT procedures within the treatment of children with ASD (Koegel & Koegel, 2006). Therefore, the PRT fidelity of treatment implementation sheet will be completed for 3 treatment sessions in the robot-based PRT condition and the PRT condition. For both conditions, a trained PRT specialist will determine fidelity by observing videos of treatment sessions. Categories of treatment fidelity are *child attending, providing a clear opportunity, interspersing maintenance and acquisition tasks, using multiple cues, follow child's choice, contingent reinforcement, natural reinforcement, reinforcement contingent on attempts*. Each category is scored within 10 1-minute intervals with a + (PRT component is utilized), - (PRT component not demonstrated) or N/A (child does not have the appropriate level or cannot be scored because the scorer is not familiar with the child). Fidelity is attained when each of the eight PRT categories are performed correctly by the clinician for 80% of the time during the intervals.

- Hours of treatment

In the current study, the care-as-usual condition includes treatments with various levels of intensity. For the robot-based PRT condition and PRT condition, the number of hours therapy is added to the number of hours care-as-usual (psycho-education sessions and medical management appointments) during the 20 weeks of intervention. For the care-as-usual condition, the number of hours care-as-usual

treatment is calculated. To account for possible effects of treatment intensity, the number of hours is provided during the course of the study will be included as a covariate in the analyses.

8.2. Randomisation, blinding and treatment allocation

.When subjects are meeting the inclusion criteria, they are randomly assigned to one of two intervention conditions, depending on the treatment location. For 4 locations of Karakter, subjects are randomized between the robot-based PRT and care-as-usual (ratio 2:1) and for 3 locations of Karakter, subjects are randomized between PRT and care-as-usual (ratio 2:1). Randomization is done by the principal investigator of the study (prof. dr. J. K. Buitelaar). Since an intention-to-treat approach is used, the randomization code is not broken if a subject drops out or shows non-compliance with the study. The study is open-labeled, because both the subjects and therapists know to which treatment condition the subject is assigned. However, the rating of the CGI-I is blinded, because this is rated by observing a video of the interaction between the child and a student and not during a treatment session.

8.3. Study procedures

Figure 4 (see Attachment 3) provides an overview of the procedures and measures that will be administered in the subjects and their caregivers. Therapist or treatment coordinators that are extensively informed about the study protocol will inform parents of possible subjects about the study. At first, parents of possible subjects (based on information in case files) that will start on treatment for ASD will be contacted. If this provides an insufficient number of subjects, parents of patients that are already receiving treatment for ASD are contacted. Parents of possible subjects will receive an information letter regarding the aim, outline and time investment of the study and will receive and informed consent form (see additional files). When parents agree with the participation of their child in the study, both parents are asked to sign and return the informed consent form. Additionally, a teacher or attendant of the child will receive an information letter on what is expected from them during the study. After obtaining informed consent from both parents, initial measures are administered to further assess inclusion criteria (inclusion and exclusion criteria are described in detail within section 3). Initial measures are:

Demographic information regarding gender, age, and language spoken by parents, diagnosis of ASD, comorbid conditions and medication use: this is determined by analyzing case files.

Confirmation of ASD diagnosis: when not administered within two years before start of the study, the ADOS-2 (De Bildt et al., 2013) is administered as an initial measure for confirming diagnosis of ASD. Besides confirmation of ASD diagnosis, the initial administration of the ADOS is used as a baseline measure in the current study. The content of the ADOS is described in section 8.1.1.

Intelligence quotient (IQ): if no measure for estimating IQ is administered within two years before the start of the study, an intelligence scale is included as an initial measure for confirming the inclusion criterion of TIQ > 70. Depending on the age and appropriateness for the child, the WISC (Kort et al., 2005), WPPSI (Hendriksen & Hurks, 2009) or MSEL (Mullen, 1995) are administered. Depending on the version that is used, the duration of the administering the scale for measuring IQ varies from 30 – 150 min.

When children meet all the inclusion criteria, they will be selected for participation in the study and will be cluster-randomly assigned to one of the three intervention conditions by the principle investigator of the study (prof. dr. J. K. Buitelaar).

Baseline

Before start of the intervention, baseline measures are administered. In all 3 conditions, one of the parents of the child and the child's teacher or attendant completes a digitalized version of the SRS. One of the parents also completes the digitalized version of the OBVL. A link is send to the e-mail address of the parent and teacher, in which they are asked to complete the questionnaire(s) within 1 week, but preferably as soon as possible. If the questionnaires are not completed within 6 days, parents and teachers are contacted by telephone. For parents, the possibility is provided to complete the digitalized questionnaire on a computer of Karakter (after or in parallel to a therapy appointment).Within the same week as the SRS and OBVL, the CGI-I scale is completed for each child by a child psychiatrist of Karakter, by watching a video of an interaction situation with the child (no treatment session). The duration of the interaction is max. 10 min. Within the robot-based PRT and PRT conditions, the first PRT screening scenario is administered to assess the communicative skills (CS) of the child that are targeted by the PRT. For the children in the robot-based PRT condition, the baseline session starts with an introduction of the robot, before administering the PRT screening scenario for assessing CS.

Intervention (including evaluation)

The intervention phase of the study will include 20 sessions for the robot-based PRT condition and PRT condition. While total duration of the treatment of children in the care-as-usual condition is varying, outcome measures are administered in parallel with the robot-based PRT and PRT condition. At 10 and 20 weeks of intervention within the 3 conditions, the SRS, OBVL and CGI-I are administered using similar procedures as during baseline. Before and after session 1, 10, and 20 in the PRT conditions, salivary samples are collected from the children using earlier described procedures (see 8.1.2). After session 20, the ADOS is administered as an evaluation measure of ASD symptoms. Additionally, in the robot-based PRT condition and PRT condition, the child's CS are assessed at 10, and 20 weeks with including a screening scenario within the treatment session and an interaction session after the treatment session. In the robot-based PRT and PRT condition, the percentages of learning moments (LM) on which the child shows appropriate behaviour is estimated after 3 sessions by watching a video of the session. In the children in the robot-based PRTcondition, a VAS line is administered, assessing the likability of the robot by the child (after each session involving the robot) and the affect of the child (before and after each session involving the robot). Additionally, after each treatment session involving the robot, parents are complete the Session Rating Scale about how they evaluate the treatment session and whether they evaluate the robot as useful in the session. Also, the load for parents of all participants regarding the measurements and investment in the robot-based PRT and PRT is determined at the evaluation (week 20 of the intervention).

Follow-up

At 3 months after the end of the intervention phase, the SRS, OBVL and CGI-I are administered in the 3 conditions using the same procedures as during baseline and the intervention phase. In addition, in the robot-based PRT and PRT conditions, the child's CS are assessed with a final screening session. Because there are no restrictions anymore regarding treatment indications after the end of the intervention phase, this will be a naturalistic follow-up.

8.4. Withdrawal of individual subjects

Subjects can leave the study at any time for any reason if they wish to do so without any consequences. The investigator can decide to withdraw a subject from the study for urgent medical reasons. This is done in consultation with the responsible child- and adolescent psychiatrist of Karakter. The researcher coordinating the study will have contact with the responsible therapist at least once a week.

8.4.1. Specific criteria for withdrawal (if applicable)

A subject may withdrawal from the treatment when the treatment provides significant stress for the child or parent that outweighs the benefits or when medication dosages should be changed during the study for optimizing mental or physical health of the child. However, due to the intention-to-treat approach, subjects who drop-out or show non-compliance are not excluded from the analyses in the current RCT.

8.5. Replacement of individual subjects after withdrawal

Individual subjects are not replaced after withdrawal due to the intention-to-treat approach.

8.6. Follow-up of subjects withdrawn from treatment

If possible, outcome and follow-up measures will be administered in subjects that drop-out or show non-compliance. Subjects that withdrawn from treatment due to stress or medical (e.g. medication) reasons are followed-up by the responsible child psychiatrist or GZ-registered psychologist of Karakter until possible side-effects are diminished.

8.7.Premature termination of the study

The study is terminated prematurely when treatment causes significant mental or physical stress in the majority of subjects and/or their parents or primary caregivers, that outweighs the benefits of the treatment. However, the benefits of the treatment within this study are expected to highly outweigh the risks.

9. SAFETY REPORTING

9.1. Section 10 WMO event

In accordance to section 10, subsection 1, of the WMO, the investigator will inform the subjects and the reviewing accredited METC if anything occurs, on the basis of which it appears that the disadvantages of participation may be significantly greater than was foreseen in the research proposal. The study will be suspended pending further review by the accredited METC, except insofar as suspension would jeopardise the subjects' health. The investigator will take care that all subjects are kept informed.

9.2. AEs, SAEs and SUSARs

9.2.1. Adverse events (AEs)

Adverse events are defined as any undesirable experience occurring to a subject during the study, whether or not considered related to the experimental intervention. All adverse events reported spontaneously by the subject or observed by the investigator or his staff will be recorded. It will be noted when a subject discontinues the RCT because of an urgent problem with the his/her mental or physical health or other adverse events. When urgent health problems occur during the treatment, the responsible health professional is contacted as soon as possible. However, it is not expected that any adverse events will happen due to participation in the study (see section 8.8. addressing benefits and risks).

9.2.2. Serious adverse events (SAEs)

A serious adverse event is any untoward medical occurrence or effect that at any dose:

- results in death;
- is life threatening (at the time of the event);
- requires hospitalisation or prolongation of existing inpatients' hospitalisation;
- results in persistent or significant disability or incapacity;
- is a congenital anomaly or birth defect;
- Any other important medical event that may not result in death, be life threatening, or require hospitalization, may be considered a serious adverse experience when, based upon appropriate medical judgement, the event may jeopardize the subject or may require an intervention to prevent one of the outcomes listed above.

The sponsor will report the SAEs through the web portal *ToetsingOnline* to the accredited METC that approved the protocol, within 15 days after the sponsor has first knowledge of the serious adverse events.
SAEs that result in death or are life threatening should be reported expedited. The expedited reporting will occur not later than 7 days after the responsible investigator has first knowledge of the adverse event. This is for a preliminary report with another 8 days for completion of the report.

It is not expected that any serious adverse events will happen due to participation in the study (see section 8.8. addressing benefits and risks).

9.3. Annual safety report

N/A

9.4. Follow-up of adverse events

All AEs will be followed until they have abated, or until a stable situation has been reached. Depending on the event, follow up may require additional tests or medical procedures as indicated, and/or referral to the general physician or a medical specialist. SAEs need to be reported till end of study within the Netherlands, as defined in the protocol.

9.5. [Data Safety Monitoring Board (DSMB) / Safety Committee]

Because the risks of participating in the study are estimated to be very low, review or advice of the DSMB or safety committee is not needed for the current study.

10. STATISTICAL ANALYSIS

10.1. Primary study parameter(s)

-Generalized social and communicative skills

The primary outcome measure in the study is the SRS. A mean is composed by the parent and teacher ratings to obtain one measure for generalization. On the total mean score of the SRS, a percentage of change over the course of treatment is calculated. A reduction in the total SRS score of more than 25% is regarded as a clinical response. Based on the SRS, two groups of responders are composed: clinically responding and clinically non-responding. Results will be analysed in an intention-to-treat analysis, including all subjects that are randomly assigned to the three conditions, regardless of dropout or non-compliance. The percentage responders of the treatment groups (independent variable, i.e. robot-based PRT, PRT, care-as-usual) will be compared by a chi-square analysis in planned comparisons: robot-based PRT versus care as usual and PRT versus care-as-usual.

-Change of patient's illness

Another primary outcomes measure is the CGI-I. Of the scale, a categorical clinical response score (responder, non-responder) is calculated. A responder is defined as "much" or "very much" improved on the CGI-I. As for the SRS, results are analyzed in an intention-to-treat analysis. To determine the relationship between group status and responder status, a chi-square analysis is conducted.

-Severity of ASD symptoms

The ADOS-2 is administered at baseline and at evaluation. A severity score is calculated based on Gotham et al. (2009) and a change score is computed comparing the ADOS administrations. A responder status is computed based on a significant decrease on the ADOS severity score. As for the SRS and the CGI-I, the relationship between group status and responder status on the ADOS is determined with conducting a chi-square analysis.

10.2. Secondary study parameter(s)

-Communicative skills (CS) during PRT-based interventions

Since this measure is only available for subjects in two of the three intervention conditions, separate analyses are conducted. For the prompt level that the child needs for each communicative skill, a score is calculated from 6 (spontaneously) to 0 (no response after highest prompt level). To compare communicative skills before and after the

interventions, a repeated measures ANOVA is conducted with group status (robot-based PRT or PRT) as a covariate.

-*Spontaneous appropriate behaviour in learning moments (LM) during PRT-based treatments*

Data on this measure is available for the robot-based PRT and the PRT condition. For 3 sessions, the number of spontaneous appropriate behaviours is divided by the total number of opportunities for showing spontaneous appropriate behaviour (i.e. learning moments in that session). Before and after scores on spontaneous appropriate behaviours are compared with a repeated measures ANOVA with group status (robot-based PRT and PRT) as a covariate.

-*child rearing pressure on parents*

An additional secondary outcome of the current study is the child rearing pressure that is experienced by parents, measured by the Dutch "Opvoedingsbelasting vragenlijst" (OBVL). A total mean score is calculated for the OBVL. A responder on this questionnaire is defined as significantly decreasing on the total means score. The relationship between group status (e.g. robot-based PRT, PRT, care-as-usual) and responder status on the OBVL is assessed with a chi-square analysis.

-*Likability of the robot by the child and child's affect during robot-based PRT sessions*

Since these qualitative measures are only conducted in one of the intervention conditions (robot-based PRTcondition), the outcomes of the VAS lines are not included in the main analyses. The aim of including the VAS line is highly descriptive, to support the usefulness of implementing a humanoid robot into treatment of children with ASD with qualitative measures, besides the mentioned quantitative measures. For both the likability of the robot and child's affect before and after each session, a mean is computer from the scores in the 14 treatment sessions and a change score is computed comparing VAS scores the first and final treatment session.

-*salivary cortisol, oxytocin and testosterone*

Samples will be analysed using competitive enzyme-based immunoassays. With an exploratory correlational analysis, cortisol levels are related to qualitative reports of child affect and oxytocin and testosterone levels are related to SRS outcomes of social and communicative skills.

10.3. Other study parameters

-Intelligence Quotient

For each subject, an estimate is provided for TIQ. As subjects are randomized and not matched over the intervention conditions, it is determined with an ANOVA whether subjects in the three groups significantly differ in TIQ. When significant differences are found, TIQ is included as a covariate in the analyses.

-*Age*

Chronological age of subjects at start of the intervention, as determined by analyzing case files, is included in the analyses as a covariate to assess whether implementation of a robot in the treatment is effective for both preschool and school-aged children.

-*PRT fidelity of treatment implementation*

For fidelity of PRT implementation in the robot-based PRT condition and PRT condition, a mean percentage for both conditions is calculated from the percentage of fidelity in the 5 treatment sessions. PRT fidelity is not included in the analyses, but described as a measure of reliability of treatment implementation.

-*Hours of treatment*

As a measure for treatment intensity for all three conditions, the number of hours of received treatment for each subject and/or caregiver is calculated. One variable is created for the three intervention conditions, including number of therapy/PRT hours + number of hours care as usual in the robot-based PRT and PRT condition and number of hours care-as-usual in the care-as-usual condition. Total hours of treatment is included as a covariate in the analyses to account for effects of different treatment intensities.

10.4. Interim analysis (if applicable)

N/A

11. ETHICAL CONSIDERATIONS

11.1 Regulation statement

The current study will be conducted according to the principles of the "Declaration of Helsinki", adopted by the 18th WMA General Assembly, Helsinki, Finland, June 1964 and amended by the 64th WMA General Assembly, Fortaleza, Brazil, October 2013, in accordance with the Medical Research Involving Human Subjects Act (WMO) and in accordance to the Guidelines for Good Clinical Practice (GCP) (CPMP/ICH/135/95 – 17th of July 1996).

Post-trial treatment can be provided to subjects in all treatment groups, since PRT, among other treatments for ASD, is available at different locations of Karakter within the Netherlands.

The proposal for the current study will be submitted to the CMO Arnhem-Nijmegen, an official METC in The Netherlands. Parents and other primary caregivers of possible subjects will not be approached before formal approval has been granted.

11.2 Recruitment and consent

Parents and/or other primary caregivers of patients within outpatient departments of Karakter that satisfy the inclusion criteria based on diagnostic and demographic information in case files are provided an information letter of the study (see attachment) after being informed about the study by their therapist or treatment coordinator. In addition to the information letter, parents will receive a letter in which they are asked to provide informed consent (see additional file E2). Informed consent will be obtained from both parents or legal caregivers. When both parents agree with the participation of their child in the study, they are asked to sign the informed consent form and return it within 2 weeks after receiving the information letter. When no response is given after 2 weeks, parents are contacted by telephone by the investigator (after having contacted the treatment coordinator) to provide additional information if needed and to answer questions. In addition, a teacher or attendant of the child will be contacted by telephone about the option for voluntary cooperation and will receive an information letter regarding the study. Information on the study will be place on the website of Karakter and in waiting rooms at the outpatient departments of Karakter.

11.3 Objection by minors or incapacitated subjects (if applicable)

The code of conduct for minors is applicable, see http://www.ccmo.nl/nl/gedragscodes.

11.4 Benefits and risks assessment, group relatedness

Patients assigned to the three conditions are all expected to benefit from their treatment. The robot-based PRTand PRT is provided in addition to care-as-usual (psycho-education and medical management, excepting changes in dosages of medication) that is provided within the outpatient departments of Karakter.

The risks of participating in the study are estimated to be very low. No adverse effects have been described in earlier studies that implemented a humanoid NAO robot within treatment of children with ASD. During all the robot-based PRT sessions, a trained PRT therapist is available that accurately addresses possible negative responses from children to the robot. During the programming, care is been taken to minimize executions of the robot that may evoke negative responses in children (e.g. irritations) including wrong pronunciations of the computer-generated voice of the robot and unnecessary rehearsals. Within all sessions, care is taken to optimize child’s motivation for the treatment.

11.5 Compensation for injury

Treatment that is provided within all departments of Karakter is insured within Karakter centre for child and adolescent psychiatry.

We wish to make a dispensation from the statutory obligation to provide insurance for the following reasons:

-the study is highly therapeutic and is expected to be highly beneficial for patients

-the risks of participating in the study are estimated to be very low

-no adverse events have been described in earlier studies with implementing a robot in the treatment of children with ASD

11.6 Incentives (if applicable)

Because the study is therapeutic and expected to be highly beneficial for subjects and their parents and/or legal caregivers, no additional incentives are provided.

12. ADMINISTRATIVE ASPECTS, MONITORING AND PUBLICATION

12.1 Handling and storage of data and documents

The data of the study are handled confidentially. The handling of personal data of subjects is in accordance with the Dutch Personal Data Protection Act (in Dutch: De Wet Bescherming Persoonsgegevens, WBP) and the guidelines for storage of personal data within the RadboudUMC and Karakter. After selection of subjects for the study, the principle investigator (prof. J.K. Buitelaar) will assign a code number to each subject. The code numbers further identify personal information of subjects and will only be accessible by all investigators that are involved in the project. The investigator will retain originals of all source documents for a period of 4 years.
In accordance with the guidelines for Good Clinical Practice, all study-related documents are archived for at least 15 years.

Responsible medical professionals will receive access to the personal source data when this is required for urgent medical reasons and parents and/or other legal caregivers are informed.

12.2 Monitoring and Quality Assurance

For the interventions based on PRT, fidelity of treatment implementation is determined for 3 sessions by a trained PRT psychologist. For the care-as-usual condition and the care-as-usual that is provided upon the robot-based PRT or PRT, the responsible child psychiatrist or GZ-registered psychologist of Karakter will monitor the course of treatment and will assure high quality of treatment. In addition, the general practitioner of the child is informed about inclusion in the study to optimize monitoring of mental and physical health during the study.

12.3 Amendments

Amendments are changes made to the research after a favourable opinion by the accredited METC has been given. All amendments will be notified to the METC that gave a favourable opinion.

12.4 Annual progress report

The sponsor/investigator will submit a summary of the progress of the trial to the accredited METC once a year. Information will be provided on the date of inclusion of the first subject, numbers of subjects included and numbers of subjects that have completed

the trial, serious adverse events/ serious adverse reactions, other problems, and amendments.

12.5 End of study report

The investigator will notify the accredited METC of the end of the study within a period of 8 weeks. The end of the study is defined as the last patient's last visit.
In case the study is ended prematurely, the investigator will notify the accredited METC within 15 days, including the reasons for the premature termination.
Within one year after the end of the study, the investigator/sponsor will submit a final study report with the results of the study, including any publications/abstracts of the study, to the accredited METC.

12.6 Public disclosure and publication policy

The results of the study will be submitted to peer-reviewed journals. Cooperating investigators from the RadboudUMC Nijmegen, Karakter centre for child and adolescent psychiatry and the Technical University Eindhoven are mentioned as a co-author in the publications.

13. STRUCTURED RISK ANALYSIS N/A

14. REFERENCES

APA. (2000). *Diagnostic and statistical manual of mental disorders (4th ed. text rev.)*. Washington, DC: Author.

APA. (2013). *Diagnostic and statistical manual of mental disorders*. Arlington, VA: American Psychiatric Publishing.

Baird, G., Simonoff, E., Pickles, A., Chandler, S., Loucas, T., Meldrum, D., & Charman, T. (2006). Prevalence of disorders of the autism spectrum in a population cohort of children in South Thames: the Special Needs and Autism Project (SNAP). *Lancet, 368*(9531), 210-215. doi: Doi 10.1016/S0140-6736(06)69041-7

Barakova, E., Gillessen, J., & Feijs, L. (2009). Social training of autistic children with interactive intelligent agents. *Journal of Integrative Neuroscience, 8*(1), 23-34. doi: Doi 10.1142/S0219635209002046

Billstedt, E., Gillberg, I. C., & Gillberg, C. (2005). Autism after adolescence: Population-based 13- to 22-year follow-up study of 120 individuals with autism diagnosed in childhood. *Journal of Autism and Developmental Disorders, 35*(3), 351-360.

Charlop-Christy, M. H., Le, L., & Freeman, K. A. (2000). A comparison of video modeling with in vivo modeling for teaching children with autism. *Journal of Autism and Developmental Disorders, 30*(6), 537-552. doi: http://dx.doi.org/10.1023/A:1005635326276 11261466

Dautenhahn, K., & Werry, I. (2004). Towards interactive robots in autism therapy: Background, motivation and challenges. *Pragmatics & Cognition, 12*(1), 1-35. doi: http://dx.doi.org/10.1075/pc.12.1.03dau

De Bildt, A., Greaves-Lord, K., & De Jonge, M. (2013). *ADOS-2: Autisme Diagnostische Observatie Schema. Handleiding*. Amsterdam: Hogrefe.

Diehl, J. J., Schmitt, L. M., Villano, M., & Crowell, C. R. (2012). The clinical use of robots for individuals with autism spectrum disorders: A critical review. *Research in Autism Spectrum Disorders, 6*(1), 249-262. doi: http://dx.doi.org/10.1016/j.rasd.2011.05.006 22125579

Duquette, A., Michaud, F., & Mercier, H. (2008). Exploring the use of a mobile robot as an imitation agent with children with low-functioning autism. *Autonomous Robots, 24*(2), 147-157. doi: DOI 10.1007/s10514-007-9056-5

Fabes, R. A., Martin, C. L., & Hanish, L. D. (2009). Children's behaviours and interactions with peers. In K. H. Ruben, W. M. Bukowski & B. Laursen (Eds.), *Handbook of peer interactions, relationships, and groups* (pp. 45-62). New York, YN: The Guilford Press.

Feil-Seifer, D., & Matarić, M. J. (2009). Toward socially assistive robotics for augmenting interventions for children with autism spectrum disorders. *Experimental Robotics, 54*, 201-210.

Ganz, M. L. (2007). The lifetime distribution of the incremental societal costs of autism. *Arch Pediatr Adolesc Med, 161*(4), 343-349. doi: 10.1001/archpedi.161.4.343

Ghezzi, P. M. (2007). Discrete trials teaching. *Psychology in the Schools, 44*(7), 667-679. doi: Doi 10.1002/Pits.20256

Gillesen, J. C., Barakova, E. I., Huskens, B. E., & Feijs, L. M. (2011). From training to robot behavior: towards custom scenarios for robotics in training programs for ASD. *IEEE International Conference on Rehabilitation Robotics, 2011*, 5975381. doi: 10.1109/ICORR.2011.5975381

Gotham, K., Pickles, A., & Lord, C. (2009). Standardizing ADOS scores for a measure of severity in autism spectrum disorders. *Journal of Autism and Developmental Disorders, 39*(5), 693-705. doi: http://dx.doi.org/10.1007/s10803-008-0674-3 19082876

Guy, W. (1976). The Clinical Global Impression Scale. In W. Guy (Ed.), *ECDEU Assessment Manual for Psychopharmacology - Revised* (pp. 218-222). Rockville, MD: US Dept. of

Health, Education and Welfare, ADAMHA, MIMH Psychopharmacology Research Brance.

Harper, C. B., Symon, J. B. G., & Frea, W. D. (2008). Recess is time-in: Using peers to improve social skills of children with autism. *Journal of Autism and Developmental Disorders, 38*(5), 815-826. doi: http://dx.doi.org/10.1007/s10803-007-0449-2 17874290

Hendriksen, J., & Hurks, P. (2009). *Wechsler preschool and primary scale of intelligence, 3th ed., Nederlandse bewerking (WPPSI-III-NL)*. Amsterdam: Pearson.

Howlin, P., Goode, S., Hutton, J., & Rutter, M. (2004). Adult outcome for children with autism. *Journal of Child Psychology and Psychiatry, 45*(2), 212-229.

Huskens, B., Verschuur, R., Gillessen, J., Didden, R., & Barakova, E. (2013). Promoting question-asking in school-aged children with autism spectrum disorders: Effectiveness of a robot intervention compared to a human-trainer intervention. *Developmental Neurorehabilitation, 16*(5), 345-356.

Kasari, C. (2002). Assessing change in early intervention programs for children with autism. *Journal of Autism and Developmental Disorders, 32*(5), 447-461. doi: http://dx.doi.org/10.1023/A:1020546006971 12463519

Kasari, C., Locke, J., Gulsrud, A., & Rotheram-Fuller, E. (2011). Social networks and friendships at school: Comparing children with and without ASD. *Journal of Autism and Developmental Disorders, 41*(5), 533-544. doi: DOI 10.1007/s10803-010-1076-x

Koegel, L. K. (2014). *Teaching the pivotal behavior of initiations to childen with autism*. Santa Barbara, CA: Koegel Autism Consultants.

Koegel, L. K., Camarata, S. M., Valdez-Menchaca, M., & Koegel, R. L. (1998). Setting generalization of question-asking by children with autism. *American Journal on Mental Retardation, 102*(4), 346-357. doi: http://dx.doi.org/10.1352/0895-8017%281998%29102%3C0346:SGOQBC%3E2.0.CO;2 9475943

Koegel, L. K., Koegel, R. L., Green-Hopkins, I., & Barnes, C. C. (2010). Brief report: Question-asking and collateral language acquisition in children with autism. *Journal of Autism and Developmental Disorders, 40*(4), 509-515. doi: http://dx.doi.org/10.1007/s10803-009-0896-z

Koegel, L. K., Koegel, R. L., Harrower, J. K., & Carter, C. M. (1999). Pivotal response intervention I: Overview of approach. *Journal of the Association for Persons with Severe Handicaps, 24*(3), 174-185. doi: http://dx.doi.org/10.2511/rpsd.24.3.174

Koegel, R. L., & Koegel, L. K. (2006). *Pivotal response treatment for autism: Communication, social, & academic development*. Baltimore, Maryland: Paul H. Brookes Publishing Co.

Koegel, R. L., Koegel, L. K., & McNerney, E. K. (2001). Pivotal areas in intervention for autism. *Journal of Clinical Child Psychology, 30*(1), 19-32. doi: http://dx.doi.org/10.1207/S15374424JCCP3001_4 11294074

Kort, W., Schittekatte, M., Dekker, P. H., Verhaeghe, P., Compaan, E. L., Bosmans, M., & Vermeir, G. (2005). *Wechsler intelligence scale for children, 3th ed, Nederlandse bewerking (WISC-III-NL). Handleiding en verantwoording*. Amsterdam: Harcourt Test Publishers/Nederlands Instituut voor Psychologen.

Kuhn, L. R., Bodkin, A. E., Devlin, S. D., & Doggett, R. A. (2008). Using pivotal response training with peers in special education to facilitate play in two children with autism. *Education and Training in Developmental Disabilities, 43*(1), 37-45.

Lerman, D. C., Volkert, V. M., & LeBlanc, L. (2007). Social behaviour. In P. Sturmey & A. Fitzer (Eds.), *Autism spectrum disorders: Applied behavior analysis, evidence, and practice* (pp. 125-150). Austin, Texas: Pro-ed.

Lourens, T., & Barakova, E. I. (2011). *User-friendly robot environment for creation of social scenarios.* Paper presented at the New challenges on bioinspired applications - Proceedings of the 4th international work-conference on the interplay between natural and artificial computation, Part II, IWINAC, La Palma, Canary Islands, Spain.

Macintosh, K., & Dissanayake, C. (2006). Social skills and problem behaviours in school aged children with high-functioning autism and Asperger's Disorder. *Journal of Autism and Developmental Disorders, 36*(8), 1065-1076. doi: DOI 10.1007/s10803-006-0139-5

Mullen, E. M. (1995). *Mullen scales of early learning: AGS edition*. Circle Pines, MN: American Guidance Service.

Myers, S. M., & Johnson, C. P. (2007). Management of children with autism spectrum disorders. *Pediatrics, 120*(5), 1162-1182. doi: DOI 10.1542/peds.2007-2362

Ozonoff, S. (1995). Reliability and validity of the Wisconsin Card Sorting Test in studies of autism. *Neuropsychology, 9*(4), 491-500. doi: http://dx.doi.org/10.1037/0894-4105.9.4.491

Palmen, A., Didden, R., & Arts, M. (2008). Improving question asking in high-functioning adolescents with autism spectrum disorders: Effectiveness of small-group training. *Autism, 12*(1), 83-98. doi: http://dx.doi.org/10.1177/1362361307085265 18178598

Pierce, K., & Schreibman, L. (1995). Increasing complex social behaviors in children with autism: Effects of peer-implemented pivotal response training. *Journal of Applied Behavior Analysis, 28*(3), 285-295. doi: http://dx.doi.org/10.1901/jaba.1995.28-285 7592145

Rice, C., Nicholas, J., Baio, J., Pettygrove, S., Lee, L. C., Braun, K. V., . . . Yeargin-Allsopp, M. (2010). Changes in autism spectrum disorder prevalence in 4 areas of the United States. *Disability and Health Journal, 3*(3), 186-201. doi: DOI 10.1016/j.dhjo.2009.10.008

Roelfsema, M. T., Hoekstra, R. A., Allison, C., Wheelwright, S., Brayne, C., Matthews, F. E., & Baron-Cohen, S. (2012). Are autism spectrum conditions more prevalent in an information-technology region? A school-based study of three regions in the Netherlands. *Journal of Autism and Developmental Disorders, 42*(5), 734-739. doi: DOI 10.1007/s10803-011-1302-1

Roeyers, H., Thys, M., Druart, C., De Schryver, M., & Schittekatte, M. (2011). *Screeningslijst voor autismespectrumstoornissen. Handleiding*. Amsterdam: Hogrefe.

Scassellati, B. (2007). How social robots will help us diagnose, treat, and understand autism. *Robotics Research, 28*, 552-563.

Scassellati, B., Admoni, H., & Matarić, M. (2012). Robots for use in autism research. *Annual review of biomedical engineering, 14*, 275-294.

Smith, T. (2001). Discrete trial training in the treatment of autism. *Focus on Autism and Other Developmental Disabilities, 16*(2), 86-92. doi: http://dx.doi.org/10.1177/108835760101600204

Smith, T., McAdam, D., & Napolitano, D. (2007). Autism and applied behavior analysis. In P. Sturmey & A. Fitzer (Eds.), *Autism spectrum disorders: Applied behavior analysis, evidence, and practice* (pp. 1-29). Austin, Texas: Pro-ed Inc.

van Heijst, B. F., & Geurts, H. M. (2014). Quality of life in autism across the lifespan: A meta-analysis. *Autism (published online)*. doi: 10.1177/1362361313517053

Ventola, P., Friedman, H. E., Anderson, L. C., Wolf, J. M., Oosting, D., Foss-Feig, J., . . . Pelphrey, K. A. (2014). Improvements in social and adaptive functioning following short-duration prt program: A clinical replication. First Posting. [Journal; Peer Reviewed Journal]. *Journal of Autism and Developmental Disorders*. doi: http://dx.doi.org/10.1007/s10803-014-2145-3

Verschuur, R., Didden, R., Lang, R., Sigafoos, J., & Huskens, B. (2014). Pivotal response treatment for children with autism spectrum disorders: A systematic review. *Review Journal of Autism and Developmental Disorders, 1*(1), 34-61.

Vismara, L. A., & Lyons, G. L. (2007). Using perseverative interests to elicit joint attention behaviors in young children with autism: Theoretical and clinical implications for understanding motivation. *Journal of Positive Behavior Interventions, 9*(4), 214-228. doi: http://dx.doi.org/10.1177/10983007070090040401

WHO. (1993). *The ICD-10 classification of mental and behavioural disorders*. Geneva: Author.

Wing, L., & Potter, D. (2002). The epidemiology of autistic spectrum disorders: Is the prevalence rising? *Mental Retardation and Developmental Disabilities Research Reviews, 8*(3), 151-161. doi: Doi 10.1002/Mrdd.10029

Attachment 1

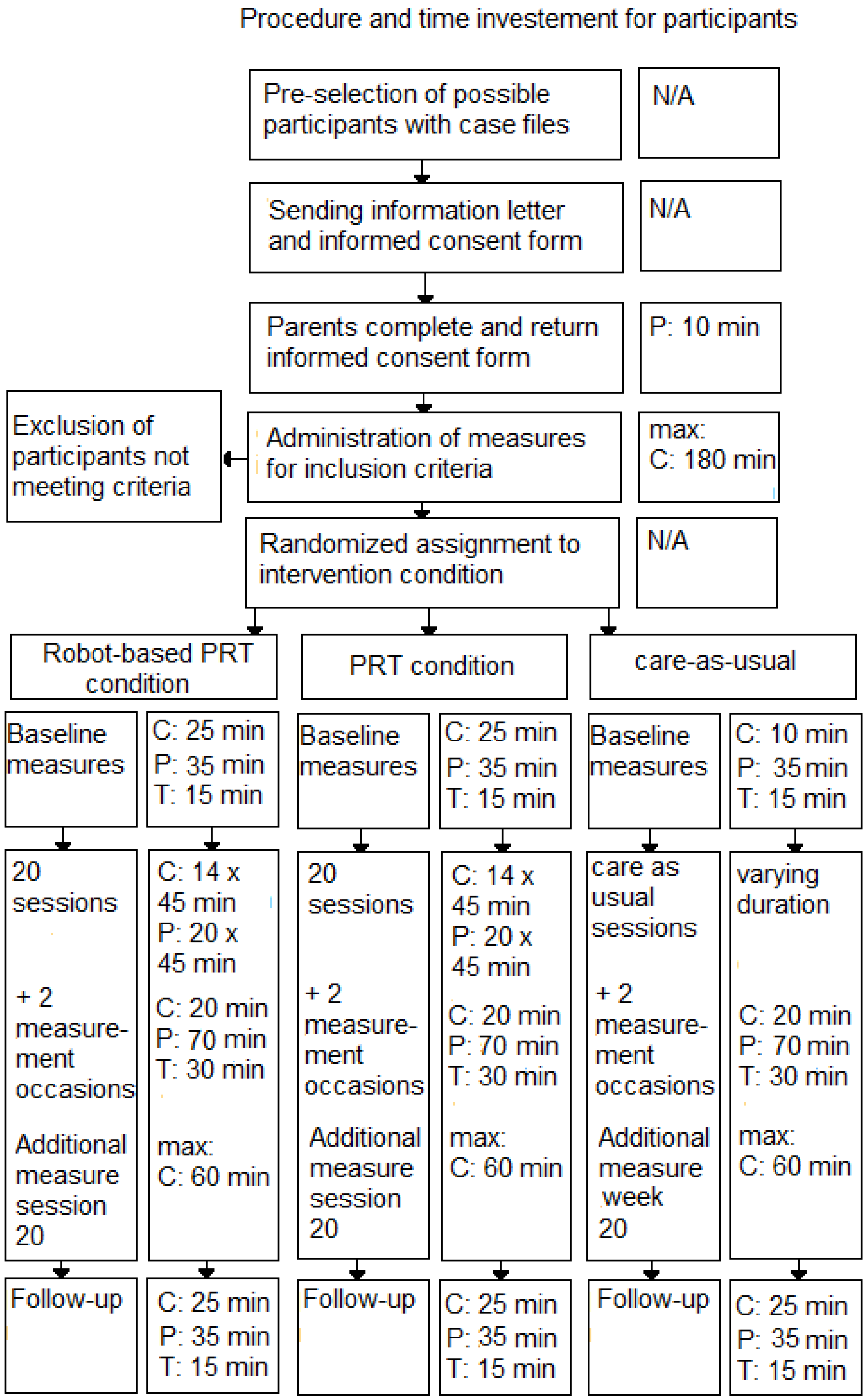

Figure 1. Overview of study procedure and time investment for subjects and their caregivers. *Note:* C = child, N/A = not applicable, P = parent, PRT = Pivotal Response Treatment, T = teacher.

Attachment 2

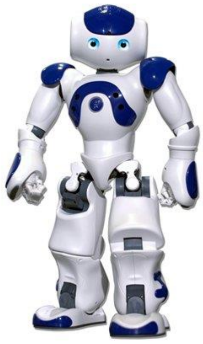

Figure 2. NAO robot from Aldebaran.

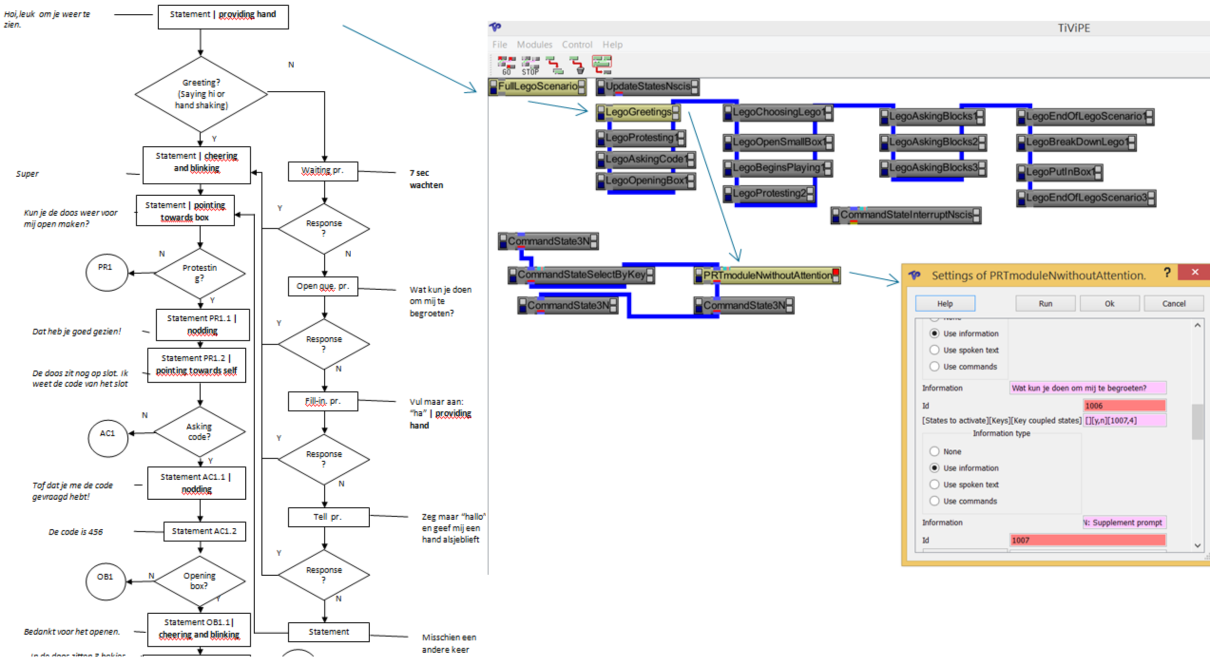

Figure 3. Implementation of a flow-chart into TiViPe.

Attachment 3

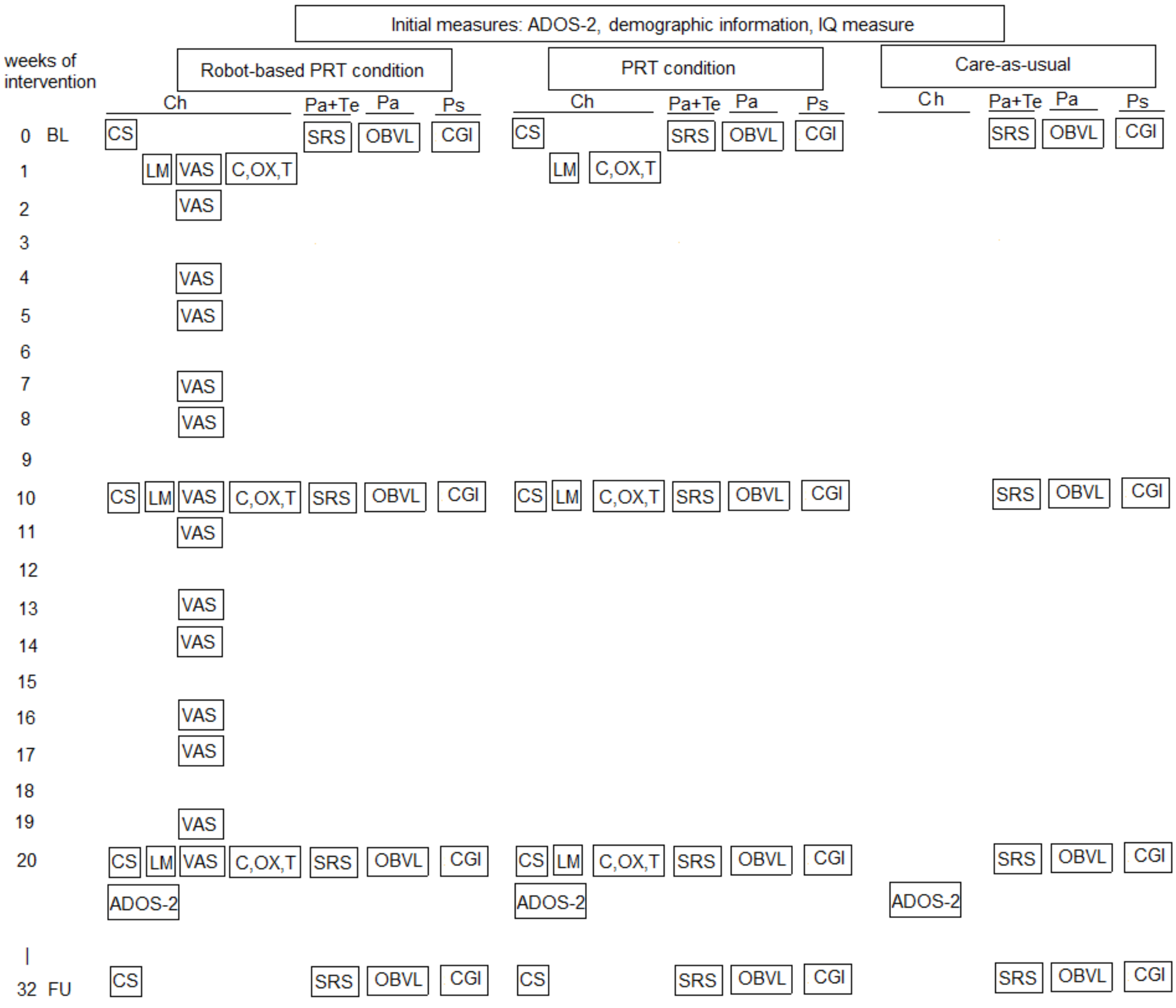

Figure 4. Overview of measures during study protocol in the three intervention groups. *Note* ADOS-2 = Autism Diagnostic Observation Schedule (2nd version), BL = baseline, C = cortisol, CGI = Clinical Global Impression, Ch = child, CS = communicative skills, FU = follow-up, IQ = intelligence quotient, LM = learning moments, OBVL = Opvoedingsbelasting Vragenlijst, OX = oxytocin, Pa = parent, PRT = Pivotal Response Treatment, Ps = psychiatrist, SRS = Social Responsiveness Scale, T = testosterone, Te = teacher, VAS = visual analogue scale.
